# Co-translational Localization of an LTR-Retrotransposon RNA to the Endoplasmic Reticulum Nucleates Virus-Like Particle Assembly Sites

**DOI:** 10.1371/journal.pgen.1004219

**Published:** 2014-03-06

**Authors:** Jung H. Doh, Sheila Lutz, M. Joan Curcio

**Affiliations:** 1 Laboratory of Molecular Genetics, Wadsworth Center, New York State Department of Health, Albany, New York, United States of America; 2 Department of Biomedical Sciences, School of Public Health, University at Albany, Albany, New York, United States of America; Fred Hutchinson Cancer Research Center, United States of America

## Abstract

The transcript of retrovirus-like transposons functions as an mRNA for synthesis of capsid and replication proteins and as the genomic RNA of virus-like particles (VLPs), wherein the genome is replicated. Retrotransposon RNA and proteins coalesce in a cytoplasmic focus, or retrosome, to initiate VLP assembly, but it is not known how the retrosome is nucleated. We determined how the RNA and Gag protein of the *Saccharomyces cerevisiae* Ty1 retrotransposon are directed to the retrosome. We found that Ty1 RNA is translated in association with signal recognition particle (SRP), a universally conserved chaperone that binds specific ribosome-nascent chain (RNC) complexes and targets the nascent peptide to the endoplasmic reticulum (ER). Gag is translocated to the ER lumen; yet, it is also found in the cytoplasm, associated with SRP-RNC complexes. In the absence of ER translocation, Gag is synthesized but rapidly degraded, and Ty1 RNA does not coalesce in retrosomes. These findings suggest that Gag adopts a stable conformation in the ER lumen, is retrotranslocated to the cytoplasm, binds to Ty1 RNA on SRP-RNC complexes and multimerizes to nucleate retrosomes. Consistent with this model, we show that slowing the rate of co-translational ER translocation by limiting SRP increases the prevalence of retrosomes, while suppressing the translocation defect of *srp* hypomorphs by slowing translational elongation rapidly decreases retrosome formation. Thus, retrosomes are dynamic foci of Ty1 RNA-RNC complexes whose formation is modulated by the rate of co-translational ER translocation. Together, these findings suggest that translating Ty1 mRNA and the genomic RNA of VLPs originate in a single pool and moreover, that co-translational localization of Ty1 RNA nucleates the presumptive VLP assembly site. The separation of nascent Gag from its RNA template by transit through the ER allows Gag to bind translating Ty1 RNA without displaying a *cis*-preference for its encoding RNA.

## Author Summary

Retrotransposons are mobile elements that have invaded the genomes of organisms from bacteria to humans. Facilitated by host co-factors, retrotransposon proteins copy their RNA genomes into DNA that integrates into the host genome, causing mutations and genome instability. The yeast Ty1 element belongs to a family of retrotransposons that are related to infectious retroviruses. Ty1 RNA and its coat protein, Gag, assemble into virus-like particles, wherein the RNA is copied into DNA. It was not previously known how Ty1 RNA and Gag are concentrated in a specific cellular location to initiate the assembly of virus-like particles. In this study, we show that Ty1 RNA is brought to the presumptive assembly site during translation by the protein chaperone, signal recognition particle. As Ty1 RNA is translated, the nascent Gag polypeptide enters the lumen of the endoplasmic reticulum, where Gag adopts a stable conformation before returning to the cytoplasm to bind to translating Ty1 RNA. An interaction between Gag molecules bound to translating Ty1 RNA results in the nucleation of the virus-like particle assembly site. Our findings identify new host co-factors in retrotransposon mobility and suggest potential approaches to controlling retrotransposon-associated genome instability in aging and cancer.

## Introduction

Long terminal repeat (LTR)-retrotransposons are ubiquitous molecular symbionts of eukaryotic genomes whose mobility is responsive to environmental and developmental cues and can result in host cell genome remodeling. These retroelements are the evolutionary progenitors of retroviruses, which have acquired *env* genes, and with them, the ability of their nucleocapsids to undergo exocytosis and infection of a naïve cell [1]. In contrast, LTR-retrotransposons lack *env* genes and replicate intracellularly. Because of their streamlined genomes and complex life cycles, both retroviruses and LTR-retrotransposons rely extensively on host cell factors to proliferate, yet much remains to be learned about the role of host cell pathways in retroelement replication.

Our understanding of the mechanism of LTR-retrotransposon replication is derived largely from the study of Ty elements in *Saccharomyces cerevisiae*. Ty1 elements comprise the most abundant and most active family of LTR-retrotransposons in budding yeast. Ty1 contains a *gag* ORF that encodes a single structural protein with capsid and nucleocapsid functions, and a *pol* ORF, which encodes protease (PR), integrase (IN) and reverse transcriptase (RT) activities. A 5.7 kb sense-strand RNA expressed from genomic Ty1 elements functions both as an mRNA and as the genomic RNA of nucleocapsids, or VLPs. Ty1 RNA is reverse transcribed in cytoplasmic VLPs to form a DNA copy (cDNA). The Ty1 cDNA is transported to the nucleus and inserted into the host cell genome by integration or more rarely, homologous recombination [2].

Ty1 RNA is translated into two precursor proteins, p49-Gag and p199-Gag-Pol, the latter a result of programmed translational frameshifting from *gag* to *pol*. The p49-Gag and p199-Gag-Pol proteins multimerize to assemble into VLPs. Gag binds Ty1 RNA and encapsidates it as a dimer into the VLP during assembly [3], [4]. Initiated by autocatalytic processing of p20-PR from the p199-Gag-Pol precursor, proteolytic processing of p49-Gag and p199-Gag-Pol to mature p45-Gag, p20-PR, p71-IN and p63-RT is thought to occur within the assembled VLP [5], [6], [7], [8].

Ty1 RNA and Gag co-localize in microscopically distinct cytoplasmic RNA foci known as T bodies [9] or retrosomes [10]. Ty1 retrosomes partially co-localize with P bodies, and many factors involved in translational repression and P body formation are also activators of Ty1 retrosome formation and retrotransposition. In fact, virtually all 5′-3′ mRNA decay and nonsense-mediated decay factors that have been analyzed are required for post-translational steps in Ty1 retrotransposition [11], [12]. Nonetheless, Ty1 retrosomes are functionally distinct from P bodies, as retrosomes disassemble during glucose starvation, which stimulates P body formation, and retrosomes are stable when translational elongation is blocked by cycloheximide, which triggers P body disassembly [9], [11]. These findings led to the idea that P body functions promote the clustering of Ty1 RNA molecules into distinct cellular foci whose formation promotes VLP assembly [10], [13]. Retrosomes are thought to be nascent VLP assembly sites because they can form in the absence of proteolytic processing of Ty1 proteins [14]. Moreover, Gag visualized by immunoelectron microscopy is found in cytoplasmic clusters that are associated with VLPs when VLP formation is induced by overexpression of Ty1 RNA [11], [15]. In P body mutants *xrn1Δ* and *lsm1Δ*, a lack of distinct Ty1 retrosomes is correlated with decreased clustering of VLPs. While the appearance of dispersed VLPs is increased in these mutants, Ty1 cDNA does not accumulate, suggesting that assembly of VLPs within the retrosome is critical for Ty1 replication [11].

Studies with plasmid-borne, galactose-inducible Ty1 (p*GAL1*:Ty1) elements in strains lacking endogenous Ty1 expression have demonstrated that retrosomes do not form when the *gag* ORF is not translated or when Gag lacking its C-terminal RNA binding domain is expressed [14], [16]. Beyond a role for functional Gag, very little is known about the requirements for the nucleation of VLP assembly sites. For example, it is not known whether Ty1 RNA is partitioned into separate pools of mRNA and genomic RNA, or whether translating Ty1 RNA can be packaged into VLPs. Moreover, the mechanism by which Ty1 RNA and Gag are directed to the presumptive VLP assembly site has not been described. One scenario that has been proposed is that Gag binds Ty1 RNA during or shortly after translation, thereby triggering its sequestration from translation. Ty1 RNA-Gag complexes could then coalesce in foci in a manner mechanistically related to the sequestration of mRNA in P bodies [10]. However, Ty1 proteins do not display a *cis*-preference for mobilizing the RNA molecule by which they are encoded [17], indicating that Ty1 Gag may be separated spatially or temporally from its RNA template after translation.

**Figure 1:**
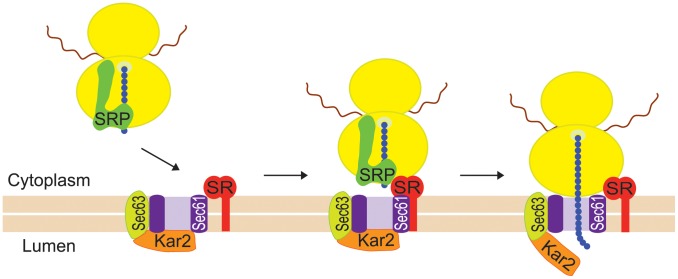
Co-translational targeting of mRNA to the ER is mediated by SRP. SRP binds with high affinity to an RNC complex via dual interactions with the ribosome (yellow ovals) and a hydrophobic sequence in the nascent peptide (blue chain). The translating mRNA is indicated by a wavy red line. SRP binding pauses translational elongation and targets the RNC complex to the ER membrane (beige bars) by interacting with the SRP receptor (SR). The SRP receptor delivers the nascent chain to the ER translocon, consisting of a channel formed by the Sec61 complex (purple shape), the associated Sec63 complex (lime green shape) and the Kar2/BiP chaperone (orange shape). SRP is released from the complex, translational elongation resumes, and the nascent peptide is translocated through the ER translocon as it is elongated.

The goal of the present study was to determine how Ty1 RNA and Gag are directed to the presumptive VLP assembly site. Specifically, we explored the hypothesis that Ty1 RNA is co-translationally localized to a specific subcellular domain, resulting in coordinated localization of Ty1 RNA and newly synthesized Gag to the presumptive VLP assembly site. Co-translational localization of mRNAs on RNC complexes is a major pathway for targeting mRNAs encoding secretory and membrane proteins to the ER so that the nascent peptides can be translocated across the ER membrane (reviewed in [18], [19]). Co-translational mRNA targeting to the ER is mediated by SRP, an evolutionarily conserved ribonucleoprotein complex that functions as a protein chaperone (reviewed in [20]). SRP has two major domains: the Alu domain, which binds the ribosome at the elongation-factor binding site and transiently pauses elongation, and the S domain, which binds a hydrophobic signal sequence in the nascent peptide. Dual binding of the ribosome and the signal domain of the nascent peptide is accompanied by a conformational change in SRP [21], and accounts for the high affinity of SRP for cognate RNC complexes [22]. SRP docks the RNC to the membrane-bound SRP receptor and aligns the nascent chain tunnel with the ER translocon, a channel through which the nascent peptide traverses the ER membrane as the mRNA template is translated (Figure 1). The ER chaperone, Kar2, interacting with the translocon-associated Sec63 complex, promotes translocation of nascent peptides to the ER lumen.

Here, we provide evidence that Ty1 RNA-nascent Gag translation complexes are associated with SRP, and nascent Gag is translocated to the lumen of the ER. Our data support a model in which Gag is folded into a stable conformation in the ER lumen and then retrotranslocated to the cytoplasm, where it binds Ty1 RNA on SRP-associated RNCs. Multimerization of Gag bound to Ty1 RNA-SRP-RNC complexes results in the coalescence of Ty1 RNA into retrosomes and likely promotes translational repression and packaging of Ty1 RNA in VLPs. These findings suggest that Ty1 RNA transitions between its role in translation and its role as the genomic RNA of VLPs. Moreover, the observation that nascent Gag is separated from its RNA template by transit into and out of the ER lumen uncovers a new mechanism by which Gag can associate with translating retroelement RNA without displaying a *cis*-preference for packaging its own RNA template.

## Results

### A defect in N-glycosylation results in Ty1 Gag instability

**Figure 2:**
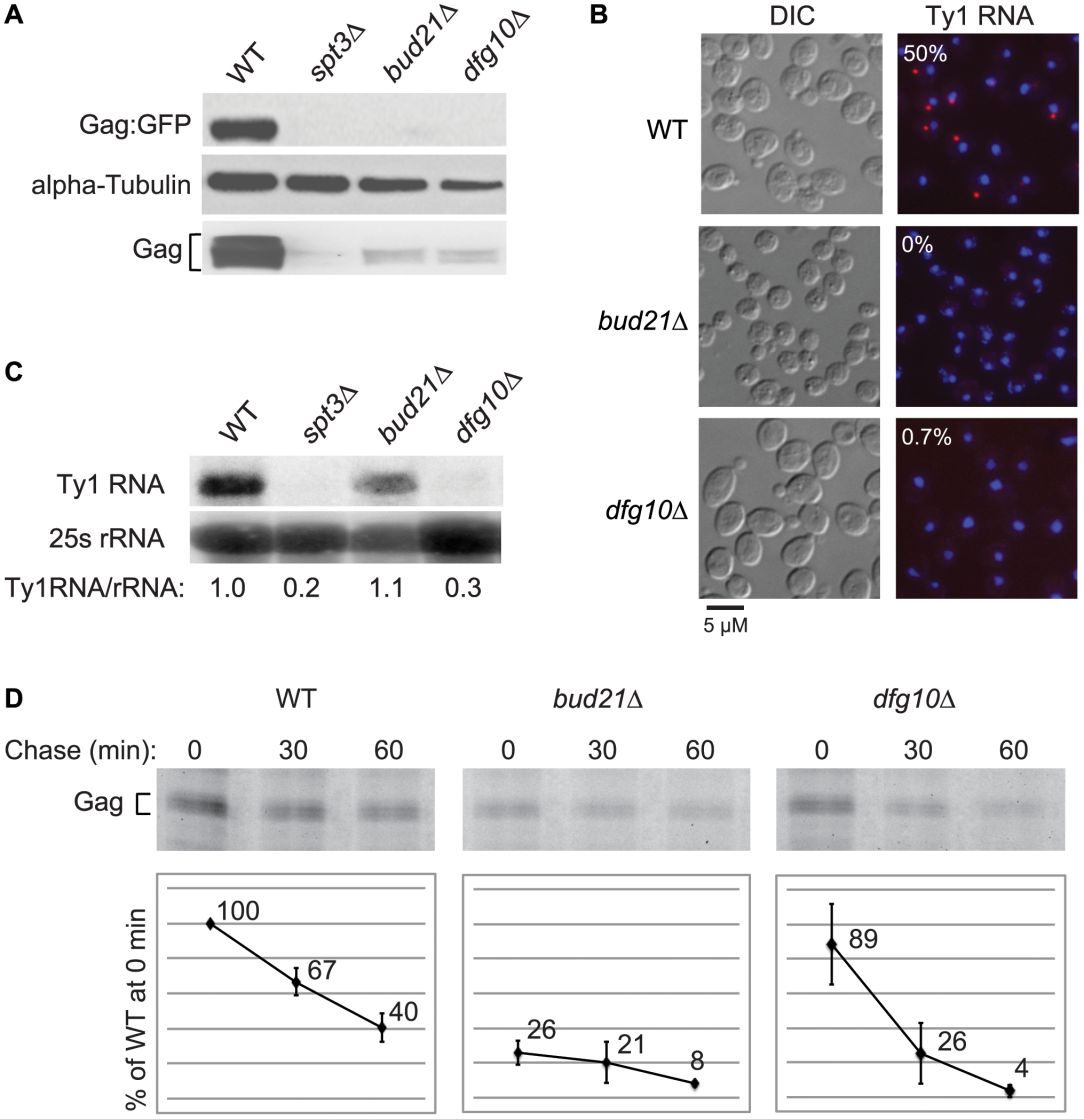
Ty1 RNA and Gag are destabilized subsequent to Gag synthesis in the N-glycosylation mutant, *dfg10Δ*. (A) Steady state levels of Ty1 Gag and Gag∶GFP relative to alpha-tubulin, a loading control, determined by western blot analysis of strain BY4741 (WT) and congenic mutant derivatives, each harboring plasmid pLTR_p_:Gag_1–401_:GFP:*ADH1*_TER_. The *spt3Δ* strain, which lacks Ty1 transcription, is a negative control. In this strain background, Gag migrates as two or more bands with faster migration than unprocessed p49-Gag (Figure S1). Multiple Gag bands may result from post-translational modification of p45-Gag. (B) FISH analysis of Ty1 RNA (red) detected using a Cy3-labeled antisense oligomer hybridizing in the Ty1 *gag* ORF. DNA (blue) was stained with DAPI, and representative merged images are presented. Cells were visualized by DIC (differential interference contrast) microscopy. The percentage of DAPI-stained cells that have one or more Ty1 RNA foci is indicated for each strain. (C) Northern blot analysis of Ty1 RNA in total RNA isolated from strains indicated. The ratio of Ty1 RNA to 25S rRNA in each strain relative to that in the wild-type strain is indicated. (D) Immunoprecipitation of Gag from cells pulse-labeled for 15 min with the methionine analog, HPG and chased with excess methionine for 0, 30 or 60 min, as indicated. HPG-labeled Gag was immunoprecipitated with anti-VLP antibody and detected by conjugation with TAMRA. TAMRA-conjugated Gag was analyzed on a 10% SDS-PAGE gel and the fluorescent signal was quantified. The values presented in the graphs are the average fluorescent signal in each strain relative to the WT strain at the 0 min chase time point. The average value from two experiments is shown. Error bars represent standard deviation.

Deletion of *DFG10*, a gene encoding polyprenol reductase, was identified in a screen for Ty1 retrotransposition-defective mutants [23]. Dfg10 catalyzes the synthesis of dolichol, the precursor for N-linked protein glycosylation in the ER. Western blot analysis of Gag protein expressed from the ∼30 endogenous Ty1 elements demonstrated that the steady-state level of Gag is substantially decreased in the *dfg10Δ* mutant (Figure 2A). A C-terminal fusion of GFP to p45-Gag (Gag:GFP), expressed from the LTR promoter on a plasmid, was also present at a reduced level in the *dfg10Δ* mutant. Ty1 RNA foci were visualized by performing fluorescent in situ hybridization (FISH) with a Cy3-labeled antisense primer in the *gag* ORF and detecting the hybrid by fluorescent microscopy. Ty1 RNA failed to efficiently localize to foci, or retrosomes, in the *dfg10Δ* mutant (Figure 2B). Similarly, deletion of the ribosome biogenesis factor gene, *BUD21*, resulted in decreased Gag, Gag:GFP and Ty1 RNA foci. To determine whether the lack of Gag accumulation in these mutants results from a reduced level of Ty1 RNA, inefficient translation or protein instability, we performed northern blot analysis of Ty1 RNA and pulse-chase labeling followed by immunoprecipitation of Gag. The steady-state level of Ty1 RNA in a *dfg10Δ* mutant was very low relative to the congenic wild-type strain or the *bud21Δ* mutant (Figure 2C). Surprisingly though, the amount of labeled Gag that was immunoprecipitated immediately after pulse-labeling of proteins in a *dfg10* mutant was 89% of that immunoprecipitated from the wild-type strain (Figure 2D, 0 min chase). In contrast, pulse-labeled Gag in the *bud21Δ* mutant was reduced to 26% of that in the wild-type strain, consistent with the proposed role for Bud21 in translation of Ty1 RNA [23]. These data indicate that synthesis of Gag is not significantly reduced by deletion of *DFG10*, despite the reduced steady-state level of Ty1 RNA. However, Gag is degraded rapidly after synthesis in the *dfg10Δ* mutant, as evidenced by the significant reduction in pulse-labeled Gag after a 30 or 60 min chase relative to the level in the wild-type strain at the same time points (Figure 2D). Thus, accumulation of Ty1 RNA and Gag is substantially reduced by a post-translational mechanism in the *dfg10Δ* mutant. The increased turnover of Gag in a mutant with a defect in N-linked glycosylation suggests that Ty1 Gag is degraded as a result of induction of the ER stress response. However, Ty1 Gag has not previously been shown to be associated with the ER.

### Gag is translocated to the ER lumen

**Figure 3:**
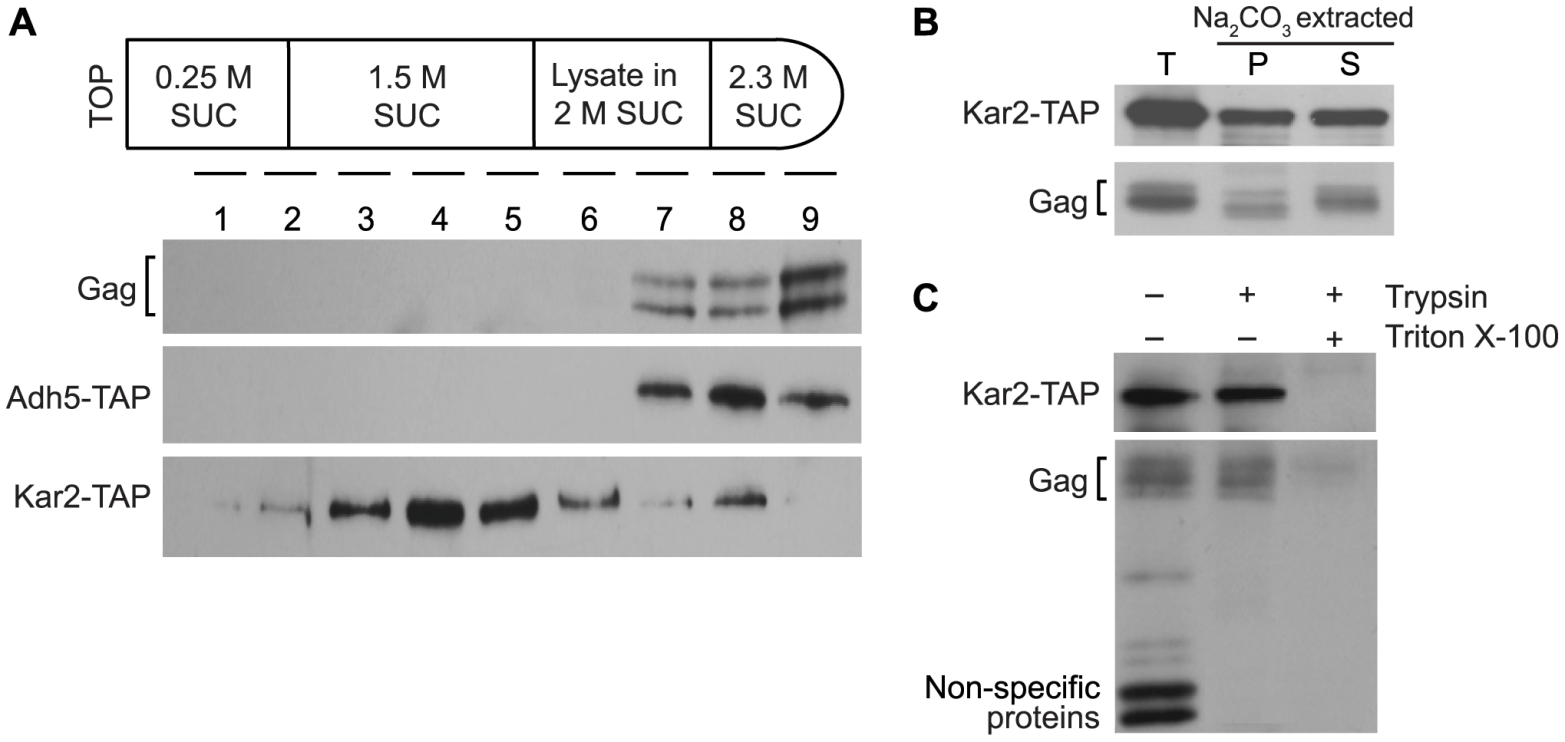
Ty1 Gag is present in the lumen fraction of ER microsomes. (A) Membrane flotation behavior of Ty1 Gag, compared to cytosolic protein Adh5 and ER lumen protein Kar2. The top panel is a schematic of the sucrose step gradients formed by adjusting cell lysate from a *KAR2-TAP* or *ADH5-TAP* strain to 2 M sucrose and loading this layer between 1.5 M sucrose and 2.3 M sucrose layers. Step gradients centrifuged at 100,000× g were fractionated from the top to the bottom of each gradient, and nine fractions of equal volume were analyzed by western blot analysis using anti-VLP polyclonal antibody to detect Gag in the *KAR2-TAP* strain, and peroxidase-anti-peroxidase complex to detect Kar2-TAP and Adh5-TAP. (B) Western blot analysis of total microsomes (T) isolated from a *KAR2-TAP* strain, and fractions from microsomes treated with sodium carbonate and centrifuged at 230,000× g to separate the membrane-associated fraction in the pellet (P) from the soluble lumen fraction in the supernatant (S). Gag was detected using anti-VLP polyclonal antibody and Kar2-TAP was detected using peroxidase-anti-peroxidase complex. (C) Western blot analysis of microsomes treated with (+) and without (−) 0.2 mg/mL trypsin in the presence (+) and absence (−) of 1% Triton X-100.

Several experiments were performed to determine whether Ty1 Gag is associated with the ER membrane or is a soluble protein in the ER lumen. To investigate the possible association of Gag with the ER membrane, equilibrium density gradient centrifugation was performed to investigate the flotation behavior of Gag (Figure 3A). To provide a membrane-associated control protein for this analysis, we used a strain in which the *KAR2* ORF, which encodes the ER chaperone, Kar2/BiP, was fused at the C-terminal end to the tandem affinity purification (TAP) tag. Kar2 is a soluble lumen protein; however, it is tethered to the membrane via interactions with the Sec63 complex and the Hrd1/Hrd3-Yos9 complex [18], [24], so Kar2 co-purifies primarily with membrane fractions [24], . Cell lysate adjusted to 2 M sucrose was loaded between higher and lower sucrose cushions in a step gradient (Figure 3A). After centrifugation of the gradient, nine fractions of equal volume were collected and analyzed by western blotting. Ty1 Gag was concentrated in high-density fractions 7 to 9. As expected, Kar2-TAP was found primarily in low-density fractions 3 to 5, but was also present in high-density fractions 7 to 9. In an identical analysis of a congenic *ADH5-TAP* strain, the cytosolic Adh5-TAP protein was found only in high-density fractions 7 to 9. The presence of Gag exclusively in high-density fractions suggests that little if any Gag associates with the ER membrane.

To determine whether Gag is present as a soluble protein in the lumen of the ER, we prepared ER microsomes from the *KAR2*-*TAP* strain. Microsomes are cell fractions enriched for closed vesicles of fragmented ER. Following disruption of microsomes with sodium carbonate, the membrane fraction was separated from the soluble lumen fraction by centrifugation. Western blot analysis was performed on total microsomes (T), as well as the membrane-enriched pellet (P) and lumen-enriched supernatant (S) fractions of sodium carbonate-extracted microsomes (Figure 3B). Ty1 Gag co-purified with total microsomes, as did Kar2-TAP. As expected, Kar2-TAP co-fractionated with both the pellet and the supernatant fractions of sodium carbonate-treated microsomes. Gag segregated primarily with the supernatant, which is indicative of its presence in the lumen of the ER. A low level of Gag was also present in the pellet fraction, which was unexpected because Gag was not apparent in low-density membrane fractions in the flotation assay (Figure 3A). The presence of Gag in the microsome pellet could result from aggregation of Gag during the incubation of microsomes with sodium carbonate. Alternatively, if a low level of Gag is transiently associated with the ER membrane, it might be detectable in these concentrated microsomal fractions as compared to the membrane fractions of the density gradient in the flotation assay.

As expected, Gag was present in microsomes prepared from the *ADH5-TAP* strain as well, but Adh5-TAP was not detected, suggesting that there was minimal contamination of microsome preparations with cytosolic proteins (unpublished result). To directly determine whether Gag association with ER microsomes is due to its presence in the ER lumen or to contamination of microsomes with cytosolic Gag, we treated the microsome preparation with trypsin (Figure 3C). If Gag is present on the outer surface of microsomes because of cytosolic contamination, it should be digested by trypsin; however, the level of Gag was not reduced by treatment with trypsin. Kar2-TAP protein was also resistant to trypsin digestion, which demonstrates that ER lumen proteins were protected in the microsome preparation. In contrast, trypsin efficiently digested small non-specific proteins, indicating that trypsin digested contaminating proteins under these conditions. When microsomes were treated with trypsin and Triton X-100, which disrupts the microsomal membrane, both Gag and Kar2-TAP were markedly reduced. Together, these findings demonstrate that some fraction of Gag is present in the lumen of the ER.

### Ty1 RNA and Gag are associated with SRP-RNC complexes

A major pathway by which proteins enter the ER is co-translational translocation mediated by SRP (Figure 1). SRP binds a hydrophobic domain of nascent peptides, including N-terminal signal sequences and transmembrane domains; however, not all peptide domains that are recognized by SRP have been defined [26]. Therefore, it is conceivable that the Ty1 Gag polypeptide, although lacking a discernible N-terminal signal sequence or transmembrane domain, could be co-translationally targeted to the ER lumen by SRP. To explore the possibility, we examined the results of a genome-wide study of SRP targets by del Alamo et al. [26]. These authors identified a collection of yeast ORFs corresponding to mRNAs that associate with SRP-RNC complexes despite the absence of a signal sequence or transmembrane domain in the corresponding nascent polypeptide. Three ORFs that encode Ty1 Gag were present in this collection: YJR027W, YBL005W-A and YGR109W-A. These findings suggest that Ty1 RNA-ribosome-nascent Gag complexes are targets of the SRP chaperone.

**Figure 4:**
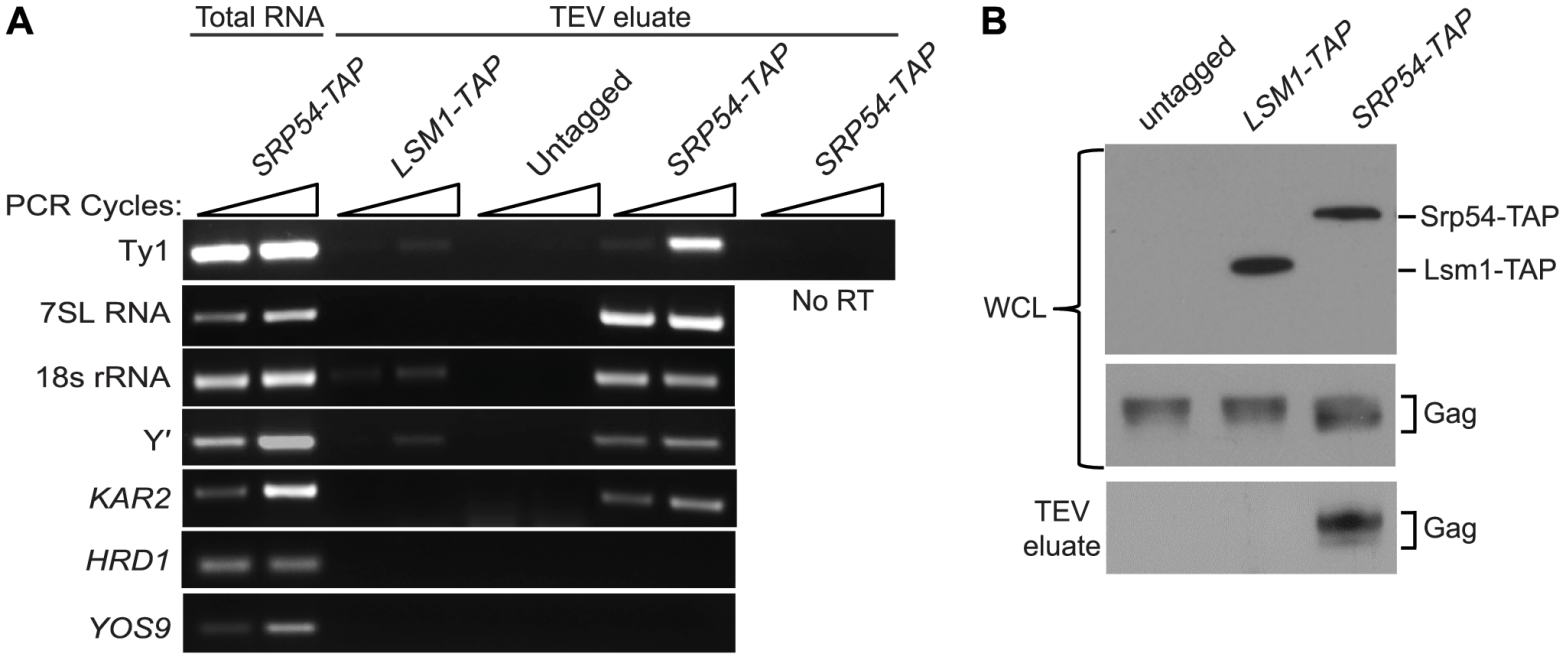
Ty1 RNA and Gag are enriched in affinity-purified SRP-RNC complexes. (A) RT-PCR analysis of RNA isolated from affinity-purified complexes from strain BY4741 (untagged) or derivatives harboring a chromosomal allele of *SRP54-TAP* or *LSM1-TAP*. Cells treated with 100 µg/ml cycloheximide to stabilize RNC complexes were immediately frozen and pulverized. TAP-tagged proteins and associated complexes were bound to IgG Sepharose and released by TEV protease. RNA isolated from TAP-purified complexes or the untagged control was reversed transcribed, and the cDNA was amplified for 29 and 32 cycles (indicated by wedge) with gene-specific primers. Reverse transcriptase was omitted from the cDNA synthesis reaction as a negative control (No RT). 7SL RNA, the RNA subunit of SRP, 18S rRNA and *KAR2* mRNA were detected as positive controls for purification of SRP-RNC complexes and *SED4* and *SEC20* mRNAs were detected as negative controls. (B) Western blot analyses of whole cell lysate (WCL) before purification using anti-CBP polyclonal antibody to detect Srp54-TAP or Lsm1-TAP and anti-VLP polyclonal antibody to detect Gag, and of TAP-purified complexes (TEV eluate) using anti-VLP antibody to detect Gag.

As an independent test of the hypothesis that Ty1 RNA is translated in association with SRP, we treated cells of an *SRP54-TAP* strain with cycloheximide to stabilize SRP-RNC complexes, affinity-purified Srp54-TAP complexes and analyzed the co-purifying RNA by RT-PCR using gene-specific primers (Figure 4A). The enrichment of SRP subunit 7SL RNA and 18S rRNA in the Srp54-TAP purification confirmed that SRP-RNC complexes were purified with Srp54-TAP. As controls for non-specific binding, cells expressing TAP-tagged Lsm1, an activator of mRNA decapping, or no TAP tag were purified under identical conditions. The lack of enrichment of 7SL RNA and 18S rRNA in the control purifications indicates that SRP-RNC complexes were specifically enriched in the Srp54-TAP purification. Next, we assayed for the association of Ty1 RNA with SRP-RNC complexes. We found that Ty1 RNA was substantially enriched in the Srp54-TAP purification, whereas its enrichment was minimal or absent in the Lsm1-TAP and no-TAP controls, respectively. Furthermore, a Ty1 PCR product was not detected in a reaction lacking reverse transcriptase, demonstrating that amplification of Ty1 sequences was not due to the presence of contaminating DNA.

Three uncharacterized ORFs, YEL076C, YLR463C and YHL049C, corresponding to the mRNA of the subtelomeric repetitive element, Y′ were also identified as interacting with SRP-RNC complexes in the genome-wide study [26]. Notably, Y′ RNA is enriched in Ty1 VLPs [27]. We found that Y′ RNA was substantially enriched in the Srp54-TAP purification but not in the Lsm1-TAP or mock purification. Similar results were obtained with *KAR2* mRNA, which is also known to interact with SRP during translation. On the other hand, mRNAs encoding the transmembrane protein Hrd1 and the ER-lumen protein Yos9, which undergo SRP-independent translation [26], were not enriched in Srp54-TAP complexes (Figure 3A). Together, these data confirm that SRP-associated RNC complexes were selectively purified in the Srp54-TAP affinity purification. Thus, we conclude that Ty1 RNA is likely translated in association with SRP and therefore, that Gag is translocated to the ER lumen during translation.

It has been proposed that binding of Ty1 Gag to translating Ty1 RNA represses its translation and promotes its packaging into VLPs [10]. Therefore, we assayed for the presence of Gag in affinity purified SRP-RNC complexes by western blotting. Gag co-purified with Srp54-TAP, but was not detected in the Lsm1-TAP or no TAP-tag purification (Figure 4B). The co-purification of Gag with SRP-associated translation complexes indicates that Gag is present not only in the ER lumen but also in the cytoplasm. Moreover, the results suggest that Gag binds Ty1 RNA that is being translated on SRP-RNC complexes. However, it is also possible that Gag bridges an interaction between non-translating Ty1 RNA and SRP-RNC complexes. To rule out this possibility and support the conclusion that Ty1 RNA is translated in association with SRP, we determined whether Ty1 RNA is associated with SRP-RNC complexes in the retrotransposition-defective *rpl7aΔ* mutant [23]. In this mutant, Ty1 RNA and Gag fail to co-localize in retrosomes, even though Ty1 RNA and Gag are present at wild type or modestly reduced levels, respectively (Figure S2 and unpublished data). These observations suggested to us that the interaction between Ty1 RNA and Gag is disrupted in the *rpl7aΔ* mutant. (The retrotransposition defect of the *rpl7aΔ* mutant will be described in detail elsewhere). We found that Ty1 RNA co-purified with 7SL RNA, 18S rRNA and Y′ RNA in Srp54-TAP complexes from the *rpl7aΔ* mutant (Figure S2A); however, no Gag was detected in the Srp54-TAP complexes (Figure S2B). In contrast, Gag co-purified with Srp54-TAP in the *bud21Δ* mutant, despite the low level of Gag in this mutant (Figure S2B). Thus, Ty1 RNA is present in SRP-RNC complexes even when Gag is not detectably associated, and therefore, Gag does not bridge the interaction between Ty1 RNA and SRP. Together, these results are consistent with a model in which Gag binds Ty1 RNA that is translated in association with SRP. Notably, the lack of Gag association with Ty1 RNA on SRP-RNC complexes in the *rpl7aΔ* mutant is correlated with an absence of Ty1 RNA and Gag localization in retrosomes (Figure S2C), raising the possibility that Gag binding to Ty1 RNA translation complexes is required for the nucleation of retrosomes.

### Depletion of co-translational ER translocation factors reduces Gag stability and Ty1 retrotransposition

**Figure 5:**
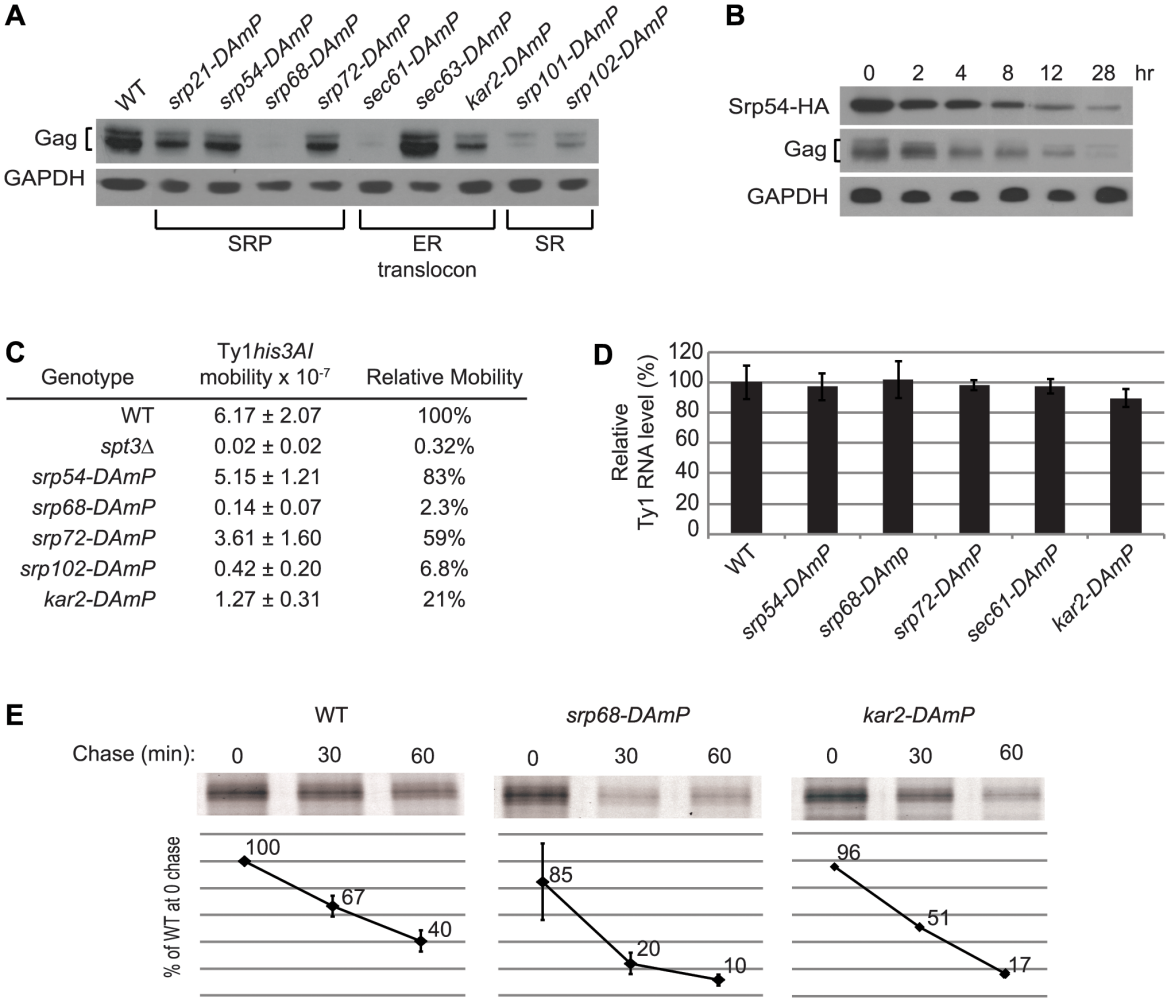
Co-translational ER translocation machinery is required for Ty1 Gag stability. (A) Western blot analysis of Ty1 Gag in strain BY4741 and the hypomorphic mutant derivatives indicated, using anti-VLP polyclonal antibody to detect Gag and anti-GAPDH monoclonal antibody to detect GAPDH as a loading control. (B) Western blot analysis of an *srp54Δ* strain expressing *GAL1p:SRP54-HA* after shutting off the *GAL1* promoter by addition of glucose to the medium. Lysate from cells removed from 0 to 28 hr after glucose addition was analyzed using anti-HA monoclonal antibody to detect Srp54-HA, anti-VLP polyclonal antibody to detect Gag, and anti-GAPDH monoclonal antibody to detect GAPDH. (C) Frequency of retrotransposition of a chromosomal Ty1*his3AI* element in strain JC3212 and hypomorphic mutant derivatives, as indicated. When the Ty1*his3AI* element undergoes retrotransposition, the intron in the *his3AI* indicator gene is spliced from the Ty1*his3AI* transcript, resulting in integration of a Ty1*HIS3* cDNA that confers a His^+^ phenotype. The frequency of retrotransposition is the frequency of His^+^ prototroph formation in each strain following growth in rich media at 20°C, the permissive temperature for retrotransposition. (D) Bar graph of the relative level of Ty1 RNA in three biological replicates of strain BY4741 and mutant derivatives, as determined from three independent Northern blots. The ratio of Ty1 RNA to 25S rRNA in each RNA sample was determined, and the mean ratio in each strain relative to the wild-type strain is expressed as a percentage. The average relative Ty1 RNA level determined from three experiments is shown. Error bars represent standard deviation. (E) Immunoprecipitation of HPG-labeled Gag from cells pulse-labeled for 15 min with HPG and chased with excess methionine for 0, 30 or 60 min, as indicated. HPG-Gag was immunoprecipitated with anti-VLP antibody and detected by conjugation to TAMRA. TAMRA-conjugated Gag was analyzed on a 10% SDS-PAGE gel and the fluorescent signal was quantified using a Typhoon scanner. The values presented in the graphs are the average fluorescent signal in each strain relative to the WT strain at the 0 min chase time point. The average value from two experiments is shown for the WT and *srp68-DAmP* strains; values presented for the *kar2-DAmP* strain are from one experiment. Error bars represent standard deviation.

The association of Ty1 RNA with SRP-RNC complexes and the presence of Gag in the ER lumen suggest that Gag is co-translationally translocated to the ER lumen. SRP directs nascent polypeptides to the ER lumen by binding to the SRP receptor and delivering the nascent peptides to the ER translocon, (Figure 1). To further explore the role of the SRP-mediated translocation pathway in the synthesis of Gag, we examined the effect of mutations in genes encoding subunits of SRP, SRP receptor, ER translocon and translocon-associated complexes on Ty1 RNA and Gag levels. Because deletion of these genes results in severe growth defects, and because Ty1 retrotransposition is heat-sensitive, we used *D*ecreased *A*bundance by *m*RNA *P*erturbation (DAmP) mutations. DAmP alleles contain a selectable marker, *KanMX*, inserted into the 3′ UTR region of the gene, which results in variable levels of mRNA destabilization and reduced gene expression [28]. To determine whether SRP-dependent translocation was compromised in the DAmP mutants, we used a phenotypic assay for translocation of a Pho8-Ura3 reporter protein to the ER [29] (Figure S3 and data not shown). The efficiency of Pho8-Ura3 translocation varied among strains carrying DAmP alleles of different co-translational translocation genes. Most mutants had partial (*srp54-DAmP*, *srp72-DAmP* and *srp101-DAmP*) or severe (*srp68-DAmP*, *sec61-DAmP* and *sec63-DAmP*) defects in Pho8-Ura3 translocation, and *sec61-DAmP* and *srp68-DAmP* mutants grew more slowly than the wild-type strain. Only the *srp21-DAmP* and *srp102-DAmP* mutants had no detectable deficiency in Pho8-Ura3 translocation relative to the wild-type strain. The relative steady-state level of Gag in each mutant was determined by western blot analysis (Figure 5A). Gag levels were reduced to varying degrees in eight mutants harboring DAmP alleles of genes encoding subunits of SRP (Srp21, Srp54, Srp68 and Srp72), the SRP receptor (Srp101 and Srp102), the ER translocon (Sec61) or the ER chaperone, Kar2. However, Gag levels were not decreased in the *sec63-DAmP* strain (Figure 5A), even though Pho8-Ura3 translocation was strongly compromised in this mutant (Figure S3). In contrast, Gag levels were markedly decreased in *srp21-DAmP* and *srp102-DAmP* mutants (Figure 5A), which lacked a detectable defect in Pho8-Ura3 translocation (Figure S3 and data not shown). Together, the data indicate that each of the nine DAmP mutants analyzed are hypomorphic; however, some of the mutations affect Gag accumulation differently than they do Pho8-Ura3 translocation. Overall, we find that Gag accumulation is reduced when co-translational ER translocation is compromised.

The results above suggest that the level of SRP could directly determine how much Ty1 Gag accumulates. To test this interpretation, we depleted functional SRP complexes by shutting off the expression of Srp54, which is required for SRP to translocate proteins to the ER [30], [31], and measuring the level of Ty1 Gag as a function of declining Srp54 levels (Figure 5B). An *srp54Δ* strain harboring plasmid pGAL1:SRP54-HA was shifted from galactose medium to glucose medium to halt transcription of the *GAL1p:SRP54-HA* cassette. Srp54-HA decreased from 0 to 28 hours after the carbon source shift, and Gag levels decreased concomitantly, whereas the level of the control protein, GAPDH was unaffected. These data indicate that the level of Gag is directly correlated with the level of functional SRP, suggesting that SRP-mediated ER translocation is necessary for the accumulation of Gag.

Depletion of Gag in mutants with hypomorphic alleles of SRP, SRP receptor and ER translocon subunit genes is correlated with a defect in retrotransposition of a chromosomal Ty1*his3AI* element (Figure 5C). The mobility of Ty1*his3AI* was measured quantitatively by determining the frequency of His^+^ prototroph formation in each strain [32]. Mutants with a small reduction in Gag accumulation, such as *srp54-DAmP* and *srp72-DAmP*, displayed an insignificant reduction in retrotransposition, while those with very low levels of Gag, such as *srp68-DAmP* and *srp102-DAmP* mutants, had a strong reduction in retrotransposition to ∼2% to 7% of that in a wild-type strain. The reduction in Gag levels and retrotransposition were not due to a decrease in Ty1 RNA levels, as the amount of Ty1 RNA in the hypomorphic *srp54*, *srp68*, *srp72*, *sec61* or *kar2* mutant was equivalent to that in the wild-type strain (Figure 5D). To determine whether these ER translocation mutants have defects in Gag synthesis or stability, we performed pulse-chase labeling and immunoprecipitation of Gag using two strains, *kar2-DAmP* and *srp68-DAmP*, which have low or undetectable steady-state levels of Gag (Figure 5E). After a 15-min pulse-label, the amount of labeled Gag detected in the *kar2-DAmP* and *srp68-DAmP* mutants was 96% or 85% of that in the wild-type strain, respectively. However, the level of pulse-labeled Gag was two or four-fold lower, respectively, after a 60 min chase in the *kar2-DAmP* or *srp68-DAmP* mutant relative to the wild-type strain (Figure 5E). The data demonstrate that Gag is synthesized efficiently but degrades more rapidly when ER translocation is compromised. We conclude that translocation to the ER is necessary for the stability of Gag. Possibly the oxidizing environment of the ER lumen enables Gag to adopt a stable conformation, or Gag may undergo post-translational modification in the ER. Since Gag also associates with SRP-RNC complexes in the cytoplasm (Figure 4B), the most plausible scenario to explain these findings is that Gag, once it folds into a stable conformation in the ER lumen, is retrotranslocated to the cytoplasm, where it associates with Ty1 RNA on SRP-RNC complexes.

### Retrosomes accumulate when ER translocation is blocked by treatment with tunicamycin

**Figure 6:**
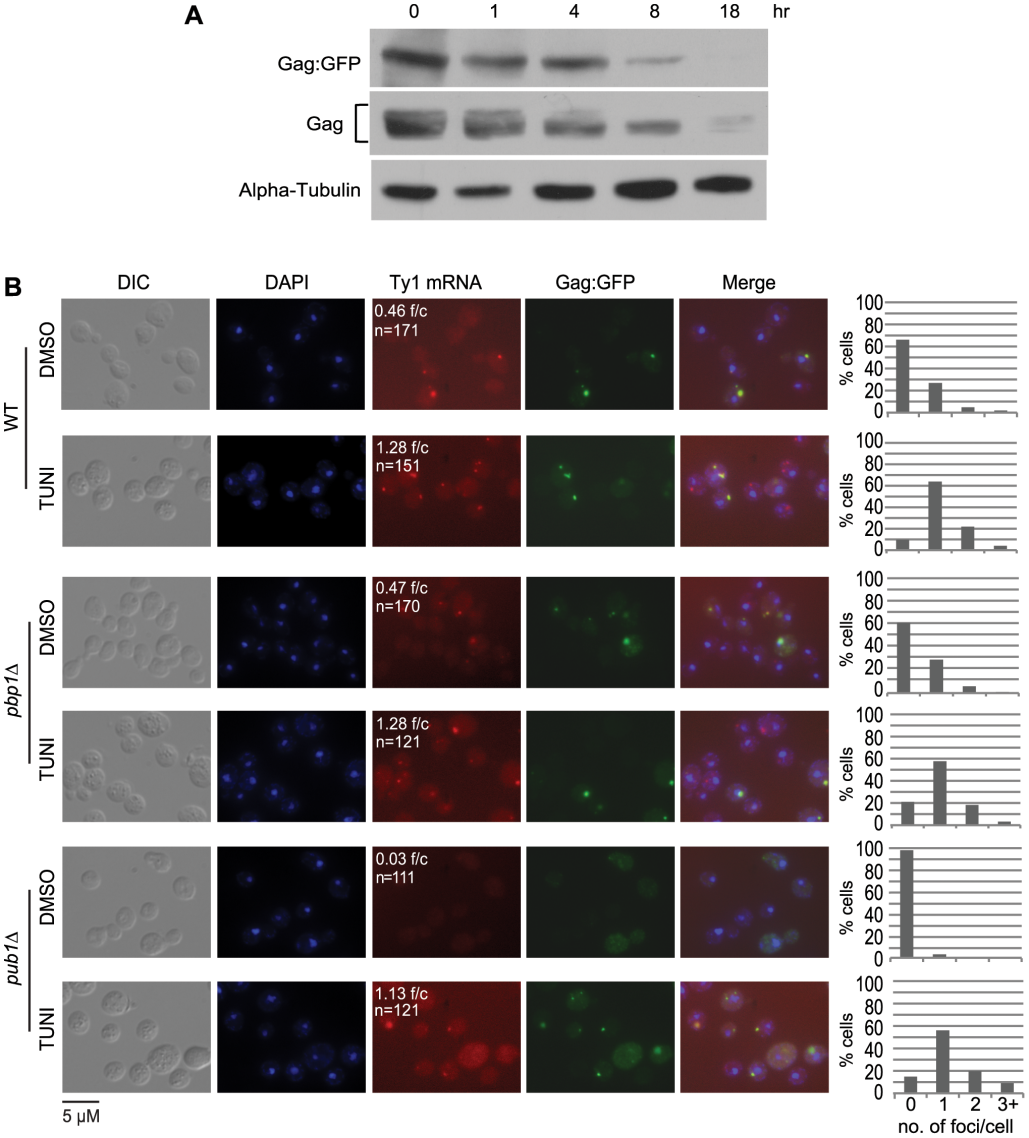
The prevalence of Ty1 retrosomes increases when ER translocation is blocked with tunicamycin. (A) Western blot analysis of strain BY4741 harboring plasmid pLTR_p_:Gag_1–401_:GFP:*ADH1*_TER_ after addition of tunicamycin to a final concentration of 5 µg/ml and incubation for the times indicated above each lane. Gag∶GFP was detected with anti-GFP antibody, endogenous Gag was detected using anti-VLP polyclonal antibody, and alpha-tubulin was detected as a loading control using anti-alpha tubulin monoclonal antibody. (B) FISH analysis of Ty1 RNA and direct visualization of Gag∶GFP in strain BY4741 (WT) and congenic *pbp1Δ* and *pub1Δ* derivatives expressing plasmid pLTR_p_:Gag_1–401_:GFP:*ADH1*_TER_ after addition of tunicamycin (TUNI) to a final concentration of 5 µg/ml or an equal volume of DMSO (mock-treatment) for 8 hours. Cells were visualized by DIC (differential interference contrast) microscopy and fluorescence microscopy. DAPI (blue) stained nuclei. A Cy3-labeled *gag* anti-sense probe detected Ty1 RNA (red) by FISH. Gag∶GFP (green) in fixed cells prepared for FISH analysis was visualized directly. **f/c** is the Ty1 RNA foci per DAPI-stained cell and **n** is the total number of DAPI stained cells counted. The graphs to the right of each image indicate the percentage of cells that have 0, 1, 2, or 3 or more RNA foci per cell.

A key question raised by the findings above is how Gag-associated Ty1 RNA-SRP-RNC complexes are temporally and spatially related to retrosomes, in which Ty1 RNA and Gag assemble into VLPs. We considered the possibility that co-translational localization of Ty1 RNA to the ER membrane and binding of Gag to Ty1 RNA-SRP-RNC complexes nucleate retrosomes. To test this hypothesis, we treated cells with tunicamycin and monitored the effect on retrosome abundance. Tunicamycin is an inhibitor of N-glycosylation that induces the unfolded protein response, which, in *S. cerevisiae*, does not attenuate translation initiation but impedes ER translocation [33], [34], [35]. Our expectation, based on the phenotype of the N-glycosylation defective mutant, *dfg10Δ* (Figure 2), is that degradation of Gag would be enhanced. In accordance with this expectation, steady-state levels of Gag decreased gradually with increasing time of exposure to tunicamycin from 1 to 18 hours (Figure 6A). To determine the effect of blocking ER translocation on the formation of retrosomes, Ty1 RNA foci were visualized in cells treated with tunicamycin for 8 hours, when Gag was decreased but not completely depleted, because Ty1 RNA foci do not form in the absence of Gag (Figure 2B). FISH and fluorescent microscopy were performed to detect Ty1 RNA. The number of Ty1 RNA foci increased substantially from 0.46 foci/cell in mock-treated cells to 1.28 foci/cell in tunicamycin-treated cells (Figure 6B). Elevated accumulation of Ty1 RNA foci comprise an increase in the percentage of cells that contained at least one retrosome from 34% to 90% and an increase in the percentage of cells with two or more retrosomes from 7% to 26%. Direct visualization of Gag-GFP expressed from plasmid pLTR:Gag_1–401_:GFP:*ADH1*_TER_ showed that Gag-GFP and Ty1 RNA co-localize in virtually all cells with detectable Gag-GFP foci, consistent with the idea that the tunicamycin-induced foci are retrosomes. These findings are consistent with the hypothesis that coalescence of translating Ty1 RNA gives rise to retrosomes. However, we also considered two alternative models that are formally consistent with these results: First, Ty1 RNA bound by Gag could be recruited to stress granules during ER stress, resulting in the formation of abnormal Ty1 RNA-Gag foci; or second, treatment with tunicamycin could induce the coalescence of non-translating Ty1 RNA-Gag complexes, or VLPs, to form retrosomes.

To determine whether Ty1 RNA and Gag accumulate in stress granules when cells are treated with tunicamycin, we measured the induction of Ty1 RNA-Gag foci by tunicamycin in strains lacking stress-granule components Pub1 and Pbp1 (Figure 6B). Pub1, an ortholog of mammalian TIA-1, and Pbp1, an ortholog of mammalian ataxin-2, are both required for stress granule assembly in yeast and mammalian cells [36], [37], [38]. The absence of Pbp1 had no effect on the prevalence of Ty1 RNA foci or the co-localization of Gag in mock-treated cells or tunicamycin-treated cells. In the *pub1Δ* mutant, Ty1 RNA-Gag foci were absent from mock-treated cells, which is likely a consequence of the reduced Ty1 RNA stability and Gag levels in this mutant [39], [40]. However, treatment with tunicamycin for 8 hours completely suppressed the Ty1 RNA localization defect of the *pub1Δ* mutant, and remarkably, restored Ty1 RNA foci to levels approaching those in wild-type cells treated with tunicamycin. Moreover, Gag co-localized with Ty1 RNA foci in tunicamycin-treated *pub1Δ* cells. These data demonstrate that neither Pbp1 nor Pub1 is necessary for the accumulation of Ty1 RNA-Gag foci in tunicamycin-treated cells, and therefore the observed Ty1 RNA-Gag foci are not stress granules. Furthermore, the tunicamycin-induced formation of retrosomes in a *pub1* mutant, which is normally depleted of Gag and therefore VLPs, strongly suggests that retrosomes are not nucleated by a coalescence of VLPs. Thus, stalled Ty1 RNA translation complexes that accumulate when ER translocation is blocked by inhibiting N-linked glycosylation likely nucleate retrosomes.

### Retrosome abundance is a function of the rate of ER translocation and Gag accumulation

**Figure 7:**
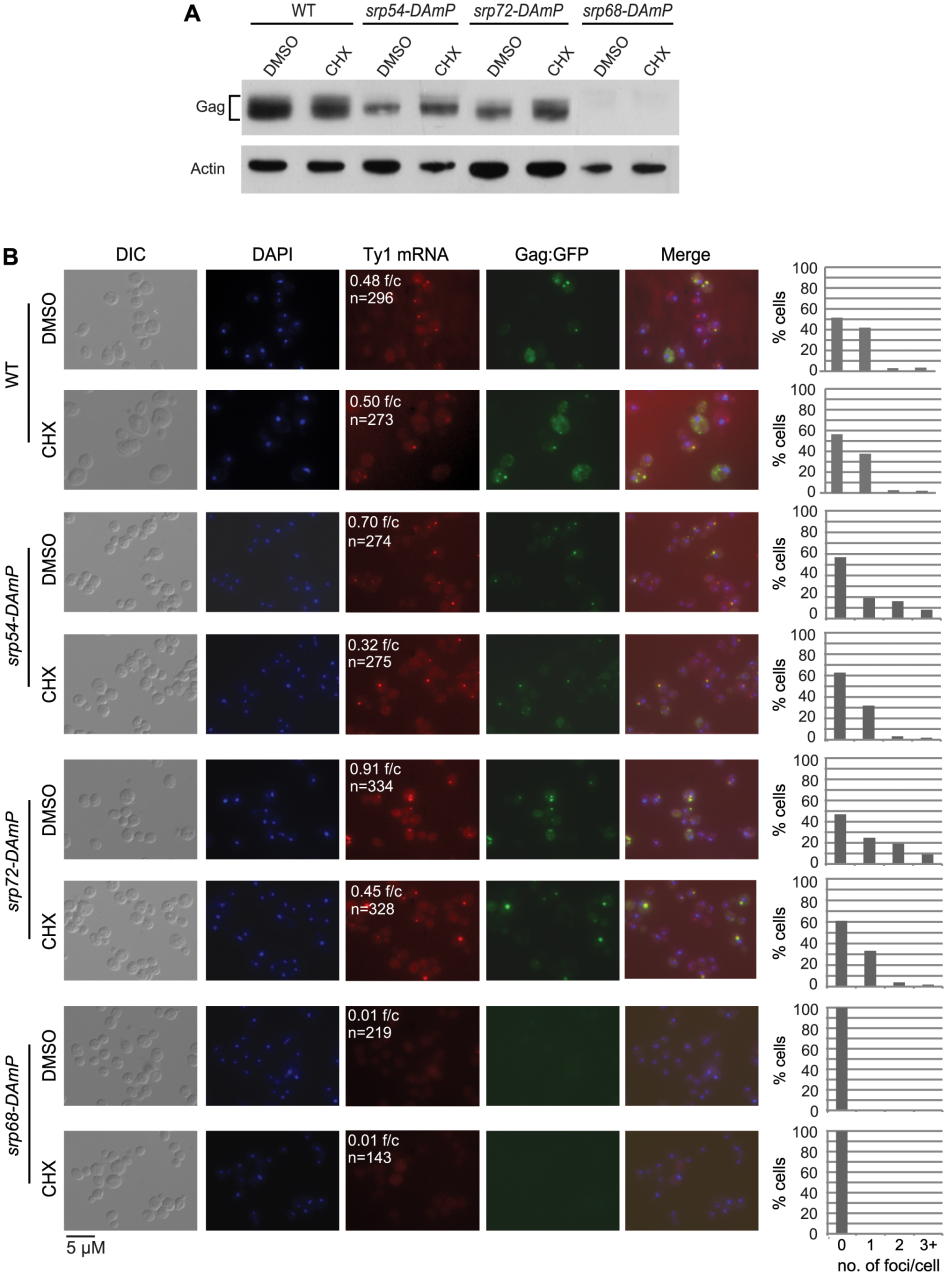
Ty1 retrosome formation is enhanced in *srp* hypomorphs and is rapidly reversed by slowing translation elongation. (A) Western blot analysis of Gag in strain BY4741 (WT) and *srp54-DAmP*, *srp72-DAmP*, and *srp68-DAmP* derivatives harboring plasmid pLTR_p_:Gag_1–401_:GFP:*ADH1*_TER_ after addition of cycloheximide (CHX) to a concentration of 0.44 µg/ml or an equal volume of DMSO (mock-treatment) for 30 min at 20°C. (B) FISH analysis of Ty1 RNA and direct visualization of Gag∶GFP in the cells described in (A). Cells were visualized by DIC (differential interference contrast) microscopy and fluorescence microscopy. DAPI (blue) stained nuclei. Ty1 RNA (red) was detected using a Cy3-labeled *gag* anti-sense probe. **f/c** is the Ty1 RNA foci per DAPI-stained cell and **n** is the total number of DAPI stained cells counted. Each graph indicates the percentage of cells that have 0, 1, 2, or 3 or more Ty1 RNA foci per cell.

As an independent test of the model that retrosomes are nucleated by an accumulation of Ty1 RNA-RNC complexes, we determined whether retrosome formation increases when ER translocation is stalled by a limited depletion of SRP (Figure 7). To examine the effect of reducing the level of SRP, we used hypomorphic alleles of *SRP54*, *SRP68* and *SRP72*, which encode components of the S domain of SRP. Depletion of these SRP subunits reduces the rate of SRP-dependent protein translocation [41]. In the *srp54-DAmP* and *srp72-DAmP* strains, which have a slight reduction in the steady-state level of Gag (Figure 7A) and a modest Pho8-Ura3 translocation defect (Figure S3 and data not shown), Ty1 RNA foci increased to 0.70 and 0.91 foci/cell, respectively, compared to 0.48 foci/cell in the wild-type strain (Figure 7B). Gag-GFP foci that were detected co-localized with Ty1 RNA foci, indicating that the foci are retrosomes. These data are consistent with the accumulation of Ty1 RNA-RNC complexes in foci when SRP is limiting, and thus translocation of Gag to the ER lumen is stalled. In contrast, the *srp68*-*DAmP* mutant, which has a very low level of Gag (Figure 7A) and a severe Pho8-Ura3 translocation defect (Figure S3), lacked Ty1 RNA foci. This phenotype is similar to that of the *bud21Δ* mutant, which also has a wild-type level of Ty1 RNA and a very low level of Gag (Figure 2). Together, the data suggest that a minimum level of Gag is needed for Ty1 RNA to coalesce in foci and support the idea that multimerization of Gag molecules bound to Ty1 RNA on SRP-RNC complexes nucleates the formation of retrosomes.

A further prediction of the model that retrosomes form by accumulation of Ty1 RNA-RNC complexes is that suppressing the translocation deficiency of the *srp54-DAmP* or *srp72-DAmP* mutant should reverse the elevated formation of retrosomes in each mutant. Treatment of *srp* hypomorphs with a very low concentration of cycloheximide suppresses their translocation deficiency by providing more time for SRP to sample RNC complexes for a cognate nascent peptide [31]. Therefore, we treated *srp54-DAmP* and *srp72-DAmP* mutants and the wild-type strain with 0.44 µg/ml cycloheximide for 30 min. Exposure to this low concentration of cycloheximide did not decrease the level of Gag in the *srp54-DAmP* or *srp72-DAmP* mutant (Figure 7A). In fact, Gag levels were slightly increased, consistent with the suppression of stalled translocation of Gag. In contrast, the number of Ty1 RNA foci decreased sharply, from 0.7 to 0.36 foci/cell in the *srp54-DAmP* strain and from 0.91 to 0.45 foci/cell in the *srp72-DAmP* strain. This effect of cycloheximide on the accumulation of retrosomes was specific for the *srp* hypomorphs, as there was no change in the number of Ty1 RNA foci/cell as a result of cycloheximide treatment of the wild-type strain. Thus, slowing down translational elongation to complement the limiting levels of SRP in the *srp54* or *srp72* hypomorph rapidly reverses the elevated formation of retrosomes. Together, the results demonstrate that retrosomes are dynamic foci formed by the coalescence of Ty1 RNA translation complexes at the ER.

## Discussion

### Translocation of Gag to the ER lumen by SRP-mediated translation of Ty1 RNA

This study revealed an unanticipated association of Ty1 RNA and Gag with the ER. Ty1 Gag co-purifies with the lumen fraction of ER microsomes and is protected from trypsin digestion by the microsomal membrane (Figure 3), providing direct biochemical evidence that Gag is translocated to the ER lumen. Although Gag is not a secreted protein and lacks a recognizable signal sequence, two lines of evidence suggest that SRP mediates the co-translational translocation of Gag across the ER membrane. First, Ty1 RNA is enriched in affinity-purified SRP-RNC complexes (Figure 4A) [26]. The enrichment of Ty1 RNA is observed even in a mutant that lacks a detectable association of Gag with SRP-RNC complexes, indicating that Gag does not bridge the association of Ty1 RNA with SRP (Figure S2). Second, disruption of ER translocation by depleting subunits of SRP, the SRP receptor, the ER translocon or the ER chaperone, Kar2/BiP, results in decreased accumulation of Gag (Figure 5A). In the translocation-deficient *srp68-DAmP* strain, which has a very low steady-state level of Gag, newly synthesized Gag is present at 85% of that in a wild-type strain but is rapidly degraded (Figure 5E). Therefore, most if not all of the nascent Gag is co-translationally localized to the ER lumen, or at least, nascent Gag that is synthesized in the cytoplasm does not contribute significantly to the cellular pool.

### A model for the nucleation of presumptive VLP assembly sites

**Figure 8:**
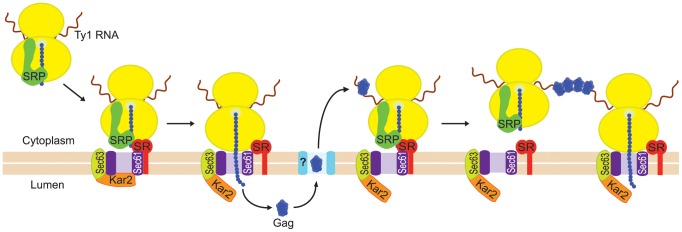
Model for nucleation of Ty1 retrosomes by coalescence of SRP-associated Ty1 translation complexes. Ty1 RNA-ribosome-nascent Gag complexes are bound by SRP, which docks the Ty1 RNA-SRP-RNC complex to the SRP receptor (SR). SRP is released from the complex, and nascent Gag traverses the ER translocon to the lumen of the ER. Gag adopts a stable conformation in the ER lumen, and then is retrotranslocated to the cytoplasm. Retrotranslocation is represented by passage of Gag through a channel (turquoise shape marked with “?”) in the ER membrane; however, nothing is known about the mechanism of retrotranslocation of Gag to the cytoplasm. In the cytoplasm, Gag binds Ty1 RNA on SRP-RNC complexes. Multimerization of Gag bound to multiple Ty1 RNAs during translation results in the coalescence of Ty1 RNA-RNC complexes into a focus that can be visualized microscopically.

Our findings support a model in which the coalescence of Ty1 RNA-ribosome-nascent Gag complexes nucleates the formation of cytoplasmic Ty1 retrosomes, where the concentration of Ty1 RNA and proteins enables the assembly of VLPs (Figure 8). In this model, Ty1 RNA-ribosome-nascent Gag complexes are specifically recognized and bound by SRP, which docks the Ty1 RNA translation complex to the SRP receptor on the ER membrane. Nascent Gag is threaded through the ER translocon into the ER lumen. Gag adopts a stable conformation in the ER lumen and is subsequently retrotranslocated to the cytoplasm. When Gag enters the cytoplasm, it binds Ty1 RNA that is being translated on SRP-RNC complexes, perhaps aided by the proximity of Ty1 RNC complexes that are docked onto the ER membrane. Multimerization of Gag bound to Ty1 RNA-SRP-RNC complexes results in the coalescence of Ty1 RNA and Gag to form the presumptive VLP assembly site. When sufficient Gag is synthesized and bound to translating Ty1 RNA, Gag likely sequesters Ty1 RNA from translation and promotes its dimerization and packaging into assembling VLPs, although the details of this transition have not yet been elucidated.

In wild-type cells, VLP assembly is inefficient, as Ty1 VLPs are rarely detected, and only about 20% Ty1 RNA is protected from nuclease digestion by encapsidation in VLPs [11], [12], [15]. Nonetheless, retrosomes are visualized in 40% to 50% of wild-type cells, suggesting that there is sufficient Gag to drive the coalescence of Ty1 RNA-RNC complexes into foci in many cells, but insufficient Gag levels to enable the efficient transition of Ty1 RNA from translation to packaging in VLPs. Thus, retrosome formation might represent a bottleneck in the retrotransposition cycle in which Ty1 RNA-SRP-RNC complexes accumulate because a component of the ER translocation machinery is limiting. This model explains why retrosomes increase in number and size when Ty1 RNA is overexpressed [11], since increasing the level of Ty1 RNA would create a larger bottleneck of Ty1 RNA-SRP-RNC complexes awaiting recognition by the SRP receptor or ER translocon.

Several lines of evidence indicate that multimerization of Gag bound to Ty1 RNA on SRP-RNC complexes results in the nucleation of retrosomes. First, Gag co-purifies with SRP-RNC complexes (Figure 4B). In an *rpl7aΔ* mutant, in which SRP-RNC complexes are associated with Ty1 RNA but not Gag, Ty1 RNA-RNC complexes fail to coalesce in retrosomes (Figure S2). Second, both the *bud21Δ* mutant, which has inefficient Gag synthesis (Figure 2D), and the *srp68-DAmP* mutant, in which Gag is rapidly degraded (Figure 5E), lack retrosomes. Thus, the level of Gag in these mutants may be too low to enable coalescence of Ty1 RNA-SRP-RNC complexes into foci. Third, modestly reducing the rate of co-translational ER translocation increases the prevalence of retrosomes. Treatment of cells with tunicamycin dramatically increased Ty1 RNA foci that co-localize with Ty1 Gag. This tunicamycin-induced accumulation of Ty1 RNA-Gag foci occurs in the absence of Pbp1 and Pub1, which are required for stress granule formation. Ty1 VLP formation is likely to be severely impaired by the paucity of Ty1 RNA and Gag in the *pub1Δ* mutant [39], [40]; therefore, the tunicamycin-induced formation of retrosomes in the *pub1Δ* mutant further supports the idea that Ty1 RNA-RNC complexes, rather than VLPs, nucleate retrosomes. The accumulation of Ty1 retrosomes that results from limiting the amount of SRP in the *srp54-DAmP* and *srp72-DAmP* hypomorphs is completely reversed by exposure of cells to a very low concentration of cycloheximide. The fact that altering the efficiency of co-translational translocation rapidly alters the prevalence of retrosomes strongly suggests that the Ty1 RNA that coalesces in retrosomes is being translated. Overall, these findings support the idea that Ty1 RNA is localized to the retrosome during translation.

We previously showed that nearly all Ty1 RNA in the cell resides in very high molecular weight complexes whose migration in sucrose gradients is not significantly altered by treatment with EDTA, which causes the dissociation of ribosomes [12]. Thus, we concluded that Ty1 RNA was translationally repressed in complexes that appeared to be devoid of translating ribosomes. However, the data presented here clearly indicate that Ty1 RNA is translated in association with SRP. Moreover, translation of Ty1 RNA within these high molecular weight complexes is consistent with the broader molecular weight distribution of Ty1 ribonucleoprotein complexes in a mutant with a defect in 40S ribosomal subunit biogenesis [39]. The fact that very little Ty1 RNA is released into low molecular weight complexes when ribosomes are dissociated by EDTA suggests that nearly all Ty1 RNA, including that being translated, is in a complex with Gag multimers.

The co-purification of Gag with SRP-RNC complexes in which Ty1 RNA is translated suggests that Gag binding to translating Ty1 RNA promotes the transition of Ty1 RNA from translation to packaging in VLPs. Thus, our findings provide indirect evidence that Ty1 RNA is not partitioned into separate mRNA and genomic RNA pools. Binding of a retroelement RNA chaperone protein to its RNA during translation is thought to enable the preferential mobilization of the encoding RNA *in cis*, which results in mobilization of only those elements that encode functional proteins. For example, human L1 retrotransposon proteins show a strong *cis*-preference for mobilizing their own RNA template, presumably because they bind to L1 RNA co-translationally. As a consequence, only functional L1 elements transpose efficiently [42], [43]. However, Ty1 proteins efficiently package Ty1 RNA *in trans*, and defective elements are mobilized as efficiently as competent elements [17], [44], raising the question of how Gag associates with translating RNA without interacting preferentially with its own RNA template. A similar paradox is manifest by studies on the nucleation of HIV-1 assembly sites: while HIV-1 mRNA and genomic RNA reside in a single pool, and Gag binds HIV-1 RNA in the cytoplasm before transport to the plasma membrane assembly site, HIV-1 Gag does not display a *cis*-preference for packaging its encoding RNA [45], [46]. Our data reveal a novel mechanism by which Gag is temporarily separated from its RNA template by translocation to the ER and retrotranslocation to the cytoplasm before binding Ty1 RNA translation complexes. This model may have implications for other retrotransposons or retroviruses that display efficient *trans*-packaging of RNA.

### The role of Gag in Ty1 RNA stability

The observation that Ty1 RNA levels are not reduced in mutants with very low levels of Gag, including the *bud21Δ* mutant (Figure 2C) and the *srp68-DAmP* mutant (Figure 5D) indicates that Gag binding to Ty1 RNA is probably not required for the exceptionally long half-life of Ty1 RNA [47]. Our conclusion that Gag is dispensable for Ty1 RNA stability differs from the conclusion reached in a recent study in which the authors analyzed a Ty1 transcript with a premature stop codon placed directly after the start codon [16]. The difference between our results may indicate that a minimal level of Ty1 RNA translation, rather than the presence of stable Gag, is required for Ty1 RNA stability. In the *dfg10Δ* mutant, Ty1 RNA is unstable (Figure 2B), although its degradation occurs subsequent to Gag synthesis (Figure 2D). Perhaps Ty1 RNA instability in the *dfg10Δ* strain is an indirect result of N-linked glycosylation, which is expected to induce unfolded protein accumulation in the ER, whereas Ty1 RNA is not destabilized in the *srp68-DAmP* strain because the cellular stress response to blocking ER translocation is distinct from that of the unfolded protein response.

### SRP-mediated translocation of Gag to the ER and retrotranslocation to the cytoplasm

The essential role of SRP in the nucleation of presumptive Ty1 VLP assembly sites is particularly interesting in light of the association of 7SL RNA with nucleocapsids of several retroviruses [46], [48], [49]. It is not known whether the 7SL RNA has a function in retroviral replication, but it has been implicated in the incorporation of the human antiviral restriction factor APOBEC3G into HIV-1 particles [50]. In addition, SRP interacts co-translationally with the Gag polyprotein of the murine endogenous retrovirus, IAP, and brings Gag to the ER membrane, although Gag was not translocated to the ER lumen in an *in vitro* system [51]. Our data raise the possibility that the association of 7SL RNA with retroviral particles evolved from an ancient functional role of SRP in retrotransposition.

The SRP-mediated translocation of Ty1 Gag to the ER lumen and the presence of Gag in the cytoplasm raises many questions about the mechanism and purpose of Gag transit into and out of the ER lumen. For example, the target sequence in the nascent Gag peptide that is recognized by SRP is not known. A systematic identification of nascent peptides that interact with SRP demonstrated that approximately 20% of SRP targets lack a predicted N-terminal signal sequence or transmembrane domain [26]. Many of these nascent peptides, including Ty1 Gag, are encoded by mRNAs that are membrane-associated and therefore are validated targets of SRP. Binding of SRP to a hydrophobic domain of the nascent peptide is required for the high affinity association of SRP with RNC complexes and for translocation [22], so it is likely that Gag has a specific target sequence that is bound by SRP. Analysis of the Ty1-H3 Gag sequence with the Kyte-Doolittle algorithm reveals that the longest hydrophobic region of Gag resides in the C-terminal end of p45-Gag from amino acid 334 to 345 (NTVAELFLDIHA). This region is predicted to form an alpha-helical domain, which could promote recognition by SRP [52], [53]. Interestingly, amino acids 341 through 346 (LDIHAI) have been shown to be critical for the formation of VLPs [54], [55].

The findings reported here suggest that Gag that is not co-translationally translocated to the ER is retained in the cytoplasm and unstable, perhaps because it cannot fold properly. What aspect of the ER lumen environment promotes the stability of Gag? One possibility is that Gag is post-translationally modified in the lumen. Although the C-terminal domain of Gag harbors five to eight potential N-glycosylation sites revealed by NetNglyc 1.0 (http://www.cbs.dtu.dk/services/NetNGlyc/) and N-GlycoSite (http://www.hiv.lanl.gov/content/sequence/GLYCOSITE/glycosite.html) algorithms, digestion of Gag with Endo H did not alter its mobility on SDS-PAGE gel (unpublished result). Thus, there is no evidence that Gag is subject to N-glycosylation. The migration of mature Gag in two bands on SDS-PAGE gels indicates that processed Gag may be subject to post-translational modification (Figure S1). Indeed, phosphorylation of Ty1 Gag in cells treated with mating pheromone has been observed previously [56]; however, there is no evidence that these modifications are linked to transit through the ER. We suggest that a more likely possibility is that the oxidizing environment of the ER supports the folding of the Gag and Gag-Pol precursors into conformations that promote their stability, enabling them to return to the cytoplasm to bind Ty1 RNA and initiate VLP assembly. Notably, maturation of the Moloney murine leukemia virus (MMLV) Gag and Gag-Pol proteins is regulated by the redox environment. Proteolytic processing of MMLV Gag and Gag-Pol proteins is constrained in infected cells exposed to a mild oxidizing agent and induced by treatment of the immature viral particles with a reducing agent [57], [58]. Perhaps folding of Ty1 Gag and Gag-Pol in the ER lumen prevents premature maturation of Gag and Gag-Pol until they are retrotranslocated to the cytoplasm, where Gag can bind Ty1 RNA and multimerize to initiate the formation of Ty1 VLPs.

How is Gag retrotranslocated from the ER lumen to the cytoplasm? Misfolded ER proteins are retrotranslocated to the cytoplasm via the ER-associated degradation (ERAD) pathway, in which transport from the ER to the cytoplasm is coupled to polyubiquitination and proteosomal degradation (reviewed in [59]). Some proteins that are known to be retrotranslocated from the ER to the cytoplasm, such as A/B toxins that enter the ER following endocytosis [60], may utilize the ERAD pathway but escape proteasome degradation. One example of a cellular protein that is retrotranslocated from the ER is the mammalian and *Trypanosoma cruzi* calcium-binding chaperone, calreticulin [61], [62]. Calreticulin is targeted to the ER lumen via the canonical SRP-mediated pathway by recognition and cleavage of its N-terminal signal sequence. A fraction of lumenal calreticulin is subsequently retrotranslocated to the cytoplasm by a process that is regulated by the concentration of calcium in the ER. Several viruses are also known to hijack the retrotranslocation pathway to promote viral assembly. For example, the N-glycosylated ORF2 capsid protein of Hepatitis E virus is retrotranslocated to the cytoplasm. In this instance, retrotranslocation is dependent on glycosylation of the ORF2 protein in the ER [63]. Further studies aimed at understanding how Ty1 Gag is recognized as an ER substrate, what conformational changes or modifications are required for Gag stability and how Gag is retrotranslocated to the cytoplasm will likely provide significant insights into the host-retrotransposon relationship as well as illuminate poorly understood aspects of SRP target specificity and protein retrotranslocation from the ER to the cytoplasm.

## Materials and Methods

### Plasmids and yeast strains

The plasmid pLTR_p_:Gag_1–401_:GFP:*ADH1*_TER_ is a *LEU2*-marked, *CEN*-based plasmid containing a Ty1 U3 promoter, 5′ UTR and *GAG* ORF from amino acid 1 to 401 (corresponding to the processed p45-Gag protein) fused to the GFP(S65T) ORF and followed by the *ADH1* terminator. The GFP(S65T)-*ADH1*_TER_ *Bam*HI-*Eag*I fragment was PCR-amplified from a DNA template derived from pFA6a-GFP(S65T)-His3MX [64]. Ty1-H3 sequence from nucleotide 238 to 1496 was PCR-amplified and fused to the GFP(S65T)-*ADH1*_TER_ fragment by PCR splicing by overlap extension (SOEing). The Ty1 5′ UTR-Gag_1–401_:GFP(S65T)-*ADH1*_TER_ was digested with *Xho*I and *Eag*I and ligated into plasmid vector pRS415 digested with *Xho*I and *Eag*I. The resulting plasmid was digested with *Apa*I and *Xho*I, and ligated to a *Apa*I-*Xho*I fragment containing the U3 region of the Ty1-H3 3′ LTR amplified by PCR with primers PJ762 and PJ763 to yield pLTR_p_:Gag_1–401_:GFP:*ADH1*_TER_. Primer PJ762 introduced an *Apa*I site upstream of the U3 sequence. PJ763 contained a single base pair mismatch that introduced an *Xho*I site at the 3′ end of the U3 sequence.

Plasmid pGAL1-SRP54-HA, carrying a *GAL1_P_*-*SRP54-HA* expression cassette on the *URA3*-based 2-micron vector, BG1805 [65], was obtained from Open Biosystems. Plasmid pMP234 [29], which harbors a *PHO5_P_-PHO8_1–82_:URA3* reporter cassette on *LEU2*-based *CEN* vector, pRS315, was obtained from Martin Pool (University of Manchester).

The *Saccharomyces cerevisiae* strains used in this study are derivatives of strain BY4741. The *spt3Δ:kanMX*, *bud21Δ:kanMX*, *dfg10Δ:kanMX*, *pbp1Δ:kanMX* and *pub1Δ:kanMX* mutant strains were obtained from Open Biosystems [66]. The *srp21-DAmP*, *srp54-DAmP*, *srp68-DAmP*, *srp72-DAmP*, *srp101-DAmP*, *srp102-DAmP*, *sec61*-*DAmP*, *sec63*-*DAmP* and *kar2-DAmP* strains were obtained from Thermo Scientific [28]. Strain JC6008 was constructed by introducing plasmid pLTR_p_:Gag_1–401_:GFP:*ADH1*_TER_ into BY4741. Strains JC6009, JC6010, JC6011, JC6155, JC6157, JC6167, JC6169, and JC6172 were obtained by introducing plasmid pLTR_p_:Gag_1–401_:GFP:*ADH1*_TER_ into *spt3Δ:kanMX*, *bud21Δ:kanMX*, *dfg10Δ:kanMX*, *pbp1Δ:kanMX*, *pub1Δ:kanMX*, *srp54-DAmP*, *srp72-DAmP*, and *srp68-DAmP* strains, respectively.

Strain BY4741 harboring a chromosomal allele of *LSM1-TAP*-*HIS3MX*, *KAR2-TAP*-*HIS3MX* or *ADH5-TAP*-*HIS3MX* strains were obtained from Open Biosystems [67]. Strain JC6177, a *SRP54-TAP* derivative of BY4741, was constructed by PCR-mediated gene disruption of BY4741 with a PCR product containing the TAP cassette flanked by sequences at the C-terminus of the *SRP54* ORF. The PCR product was amplified from genomic DNA of the *KAR2-TAP* derivative of BY4741 using primers PJ1205 and PJ1206. The *rpl7aΔ:KlURA3 SRP54-TAP* strain JC6111, was constructed by PCR-mediated gene disruption of strain JC6177 with a PCR product containing the *pGAL-I-SceI-HygB-KlURA3* cassette [68].

Strains JC3212 and the isogenic *spt3Δ:kanMX* derivative, JC5398 have been described previously [69]. Strains JC6183, JC6184, JC6185, JC6187, and JC6189 were constructed by PCR-mediated gene disruption of strain JC3212 with PCR products amplified with primers PJ1233 and PJ1234 and genomic DNA of the *srp68-DAmP* strain, primers PJ1231 and PJ1232 and genomic DNA of the *srp54*-*DAmP* strain, primers PJ1235 and PJ1236 and *srp72-DAmP* strain DNA, primers PJ1237 and PJ1238 and *srp102-DAmP* strain DNA, or primers PJ1293 and PJ1294 and *kar2-DAmP* strain DNA, respectively.

Strain JC6159 is a haploid *srp54Δ:LEU2 ura3Δ0 leu2Δ0 his3Δ1* strain carrying plasmid pGAL1-SRP54-HA [65]. The strain was constructed by PCR-mediated gene disruption of a *trp1:hisG/trp1:hisG* derivative of strain BY4743 with a *srp54Δ:LEU2* PCR product amplified with primers PJ1207 and PJ1208 and plasmid pRS405 as a template, thereby generating an *srp54Δ:LEU2/SRP54* diploid strain. Plasmid pGal-SRP54-HA was transformed into the *srp54Δ:LEU2/SRP54* diploid strain, and tetrads were dissected to obtain the segregant JC6159.

Primers used in plasmid and strain construction are provided in Table S1.

### Western blot analyses

Strains were grown at 20°C, a temperature that is permissive for Ty1 retrosome formation and retrotransposition, to mid-log phase (OD_600_ of 0.4–0.6) unless otherwise noted. Total cell lysates were prepared as described by Yarrington et al. [70] and proteins were separated on 10% SDS-PAGE gels and transferred to polyvinylidene difluoride (PVDF) membranes. The membranes were incubated in phosphate-buffered saline (PBS) plus 0.05% Tween 20 and 1% nonfat milk containing a 1∶50,000 dilution of affinity-purified anti-VLP polyclonal antibody [12] to detect Ty1 Gag, a 1∶10,000 dilution of anti-GFP polyclonal antibody (Sigma) to detect Gag∶GFP, a 1∶7,500 dilution of peroxidase-Anti-Peroxidase (PAP) soluble complex (Sigma) to detect Kar2-TAP or Adh5-TAP, a 1∶5,000 dilution of anti-calmodulin binding protein (CBP) polyclonal antibody (Millipore) to detect Srp54-TAP, or a 1∶500 dilution of anti-HA (F-7) monoclonal antibody to detect Srp54-HA (Santa Cruz Biotechnology). Subsequently, the membrane was incubated with horseradish peroxidase (HRP)-conjugated secondary antibodies and SuperSignal West Pico chemiluminescent substrate (Pierce), and then the blots were exposed to film. The PVDF membranes were stripped of antibody in 50 mM Tris-HCl (pH 7), 2% SDS, and 50 mM DTT at 70°C for 30 min and washed in 1× PBS several times. The membrane was incubated with a 1∶10,000 dilution of anti-alpha tubulin monoclonal antibody (Millipore) to detect alpha-tubulin, a 1∶7,500 dilution of anti-actin monoclonal antibody (Abcam) to detect actin, or a 1∶5,000 dilution of anti-GAPDH monoclonal antibody (Thermo Scientific) to detect GAPDH as a loading control, and bands were visualized as described above.

### Northern blot analyses

Northern blot analysis of total RNA prepared from cells grown to mid-log phase in CSM-Leu 2% Glu or YPD broth at 20°C, was performed as described previously [27]. Plasmid pJC940 DNA was used as a template to synthesize a ^32^P-labeled riboprobe to detect Ty1 RNA [12]. Plasmid pDG513, a gift of David Garfinkel, was used as a template to synthesize a riboprobe to detect 25S rRNA. Bands were quantified by phosphorimaging.

### Pulse-chase labeling and immunoprecipitation

Proteins were pulse-labeled by incubation of cells in culture with L-homopropargylglycine, an analog of L-methionine with an alkyne side chain. Strains were grown to mid-log phase in YPD broth at 20°C. 30 OD_600_ units of cells per strain were harvested and washed three times in 5 ml of CSM-Met 2% Glu media. Click-iT L-Homopropargylglycine (Life Technologies) was added to 5 ml cell resuspensions in CSM-Met broth to a final concentration of 80 µM. Cultures were incubated at 20°C with vigorous shaking, and equal OD_600_ units of cells were harvested at 0, 30 and 60 min time points. Cells were centrifuged at 1000× g for 1 min, and cell pellets were washed twice with 5 ml of chase media (CSM-Met 2% Glu+50 mM methionine).

Cell pellets were resuspended in 100 µl HB buffer [(25 mM Tris-Cl (pH7.5), 125 mM NaCl, 5 mM EDTA, 0.5% IPEGAL, Complete Mini, EDTA-free protease inhibitor cocktail (Roche)] and vortexed at 4°C for 3 min with 0.15 g of glass beads. Following addition of 5 µl of 10% SDS, the cell extract was boiled for 5 min, and 500 µl ice-cold HB buffer was added. The extract was centrifuged for 1 min at 13000× g, and 50 µl Protein A Sepharose (GE Healthcare) was added to the supernatant, which was incubated at 4°C for 4 hr. Anti-VLP polyclonal antibody was added to pre-cleared lysate and rotated at 4°C for 18 hr. 50 µl of Protein A Sepharose was added to each sample and rotated at 4°C for 30 min. Samples were washed 3 times with 500 µl IP wash buffer [50 mM Tris (pH7.5), 250 mM NaCl, 5 mM EDTA, 1% IPEGAL, 1× EDTA-free protease inhibitor cocktail (Roche)], and proteins were eluted from the Protein A Sepharose with 50 mM Glycine (pH 3.0) and equilibrated in 200 µl of 50 mM Tris-Cl (pH 8.0), 1% SDS.

Eluted proteins were precipitated by methanol/chloroform precipitation. Copper-catalyzed triazole formation click reactions were performed using Click-iT Cell Reaction Buffer Kit (Life Technologies) to conjugate TAMRA (Tetramethylrhodamine 5-Carboxamido-(6-Azidohexanyl), 5-isomer; Life Technologies) to HPG-labeled Gag. SDS loading buffer was added to each reaction, proteins were separated on 10% SDS-PAGE gels. The fluorescent signal of TAMRA-conjugated Gag was detected using a Typhoon Scanner with a 580 BP30 filter, and bands were quantified with ImageQuant TL Software (GE Healthcare).

### Membrane flotation assay

Strain BY4741 was grown to mid-log phase in YPD broth at 20°C. The cells were harvested, washed, and then resuspended in Buffer F [50 mM HEPES-NaOH pH 7.6, 150 mM NaCl, 5 mM EDTA, 1 mM dithiothreitol supplemented with Complete Mini, EDTA-free protease inhibitor cocktail (Roche)] to an OD_600_ of 10. Glass beads were added and cells were agitated on a Vortex mixer for 3 min at 4°C. The extract was removed and centrifuged twice at 300× g for 2 min at 4°C. 50 µl of the supernatant was mixed with 300 µl of 2.3 M sucrose in Buffer F, to obtain lysate in 2.0 M sucrose. This lysate was layered onto a 300 µl cushion of 2.3 M sucrose in Buffer F in a centrifuge tube. 1.5 M sucrose in Buffer F (500 µl) and 0.25 M sucrose in Buffer F (350 µl) were successively layered onto the gradient and the tube was centrifuged in a Beckman SW55 rotor at 100,000× g for 4 hr at 4°C. A 150 µl fraction was removed from the top of the gradient and discarded. Nine 150 µl fractions were collected from the top of the gradient, and protein profiles were analyzed by western blot analysis as described above.

### Microsome preparation, sodium carbonate extraction, and protease protection assay

Microsomes were prepared from a culture of strain BY4741 grown to mid-log phase in YPD broth at 20°C as described in Brodsky et al. [71]. The microsomes were resuspended in B88 buffer (20 mM HEPES, pH 6.8, 250 mM sorbitol, 150 mM KOAc, 5 mM MgOAc) to an OD_280_ of 10. Sodium carbonate extractions were performed by incubating 50 µl of microsomes with 1 ml extraction buffer [200 mM Na_2_Co_3_ (pH 11.5), 10 mM DTT, Complete Mini, EDTA-free protease inhibitor cocktail (Roche), 0.5 M sucrose, 2% glycerol] on ice for 30 min. Samples were centrifuged at 230,000× g at 4°C for 1 hr, and the pellet was dissolved in 1× SDS loading buffer. Proteins in the supernatant were precipitated by incubation on ice for 30 min with trichloroacetic acid (TCA) added to a final concentration of 10%, followed by a 10-min centrifugation at 16,060× g at 4°C. The pellet was solubilized in 1× SDS loading buffer, and proteins separated on 10% SDS-PAGE gels were analyzed by western blot analysis, as described above.

Protease protection assays were performed by incubating 50 µl of microsomes with and without 1% Triton X-100 with 0.2 mg/ml TPCK-treated trypsin from bovine pancreas (Sigma) for 15 min on ice. The reactions were stopped by the addition of 5× SDS loading buffer. Ty1 Gag and Kar2-TAP were detected by western blot analysis, as described above.

### Affinity purification of TAP complexes, RNA and protein analysis

Srp54-TAP and control complexes were purified following the procedure of del Alamo et al [26]. Strain BY4741 and derivatives harboring a chromosomal *SRP54-TAP* or *LSM1-TAP* allele were grown to an OD_600_ of 0.6–0.7 at 20°C. Cycloheximide was added to a final concentration of 0.1 mg/ml and growth was continued for 1 min at 20°C with vigorous shaking. Cells were immediately harvested by filtration onto 0.45 µm pore size nitrocellulose filters (Whatman), and cells were resuspended in 0.5 ml of ice-cold buffer A [50 mM Hepes-KOH (pH 7.5), 140 mM KCl, 10 mM MgCl2, 0.1% NP-40, 0.1 mg/ml cycloheximide, 0.5 mM DTT, Complete Mini EDTA-free protease inhibitor cocktail (Roche), 0.2 mg/ml heparin, 50 U/ml Superasin (Ambion), and 50 U/ml RNAsin (Promega)]. The cell suspension was dripped into a 50-ml conical tube containing liquid nitrogen. Frozen cells were pulverized for six cycles of 3 min at 15 Hz, using a Retsch MM301 mixer mill. Sample chambers were chilled in liquid nitrogen before each pulverization cycle. Pulverized cells were thawed and resuspended in 5 ml of buffer A. Cell debris was removed by two sequential centrifugation steps at 8,000× g for 5 min at 4°C. A 100 µl aliquot of the supernatant was removed for total RNA isolation, and a 50 µl aliquot was removed for western blot analysis. The remaining supernatant was incubated with IgG-Sepharose 6 Fast Flow (GE Healthcare) at 4°C for 2 hr. Beads were washed once in 5 ml of buffer A for 2 min and 5 times in 1 ml buffer B [50 mM Hepes-KOH (pH 7.5), 140 mM KCl, 10 mM MgCl_2_, 0.01% NP-40, 10% glycerol, 0.5 mM DTT, 10 U/ml SUPERase-In (Life Technologies), 10 U/ml RNasin, 0.1 mg/ml cycloheximide] for 1 min. Beads were resuspended in 100 µl of buffer B, and 0.3 U/ml AcTEV protease (Invitrogen) was added. The samples were incubated for 2 hr at 16°C, and the eluate was recovered. A 50 µl aliquot of the eluate was mixed with 5× SDS loading buffer, and the proteins were separated on 10% SDS-PAGE gels. Srp54-TAP and Ty1 Gag proteins were analyzed by western blot analysis, as described above. Total RNA and RNA from the eluate were isolated by sequential extraction with Phenol/Chloroform [5∶1] (Amresco), Phenol/Chloroform/Isoamyl Alcohol [25∶24∶1] (Sigma), and chloroform followed by isopropanol precipitation with 15 µg of Glycoblue (Ambion) as carrier. Each RNA sample was treated with 0.04 U/µl Turbo DNase (NEB) for 30 min at 30°C and inactivated with DNase Inactivation Reagent for 5 min at room temperature, and cDNA was synthesized using the First-Strand cDNA Synthesis Kit (Affymetrix). PCR reactions were performed using 0.5 µg of cDNA as a template with gene-specific primers. Each reaction was subject to 29 or 32 cycles of amplification. PCR products were separated by electrophoresis on a 2% agarose gel stained with ethidium bromide. The sequence of gene-specific RT-PCR primers is provided in Table S2.

### Srp54 depletion

Cells of strain JC6159 were diluted to an OD_600_ of 0.01–0.02 in CSM-Ura-Leu 2% Gal 2% Raf 2% Suc and grown at 20°C for 24 hours. Glucose was added to the culture to a final volume of 2%, and 4 OD_600_ units of cells were harvested, pelleted and frozen at different time points. Equal volumes of lysate prepared from each cell pellet were separated on 10% SDS-PAGE gels and analyzed by western blot analysis as described above.

### Transposition frequency assays

To measure transposition of the chromosomal *Ty1his3AI[Δ1]-3114* element [69], strain JC3212 and mutant derivatives were grown in YPD broth at 30°C to saturation. Cultures were diluted 1∶1000 in YPD broth and grown at 20°C for three days. 1–5 µl of a 1∶1000 dilution of each culture was plated on YPD agar to determine the colony forming units (CFU). Aliquots (1–2 ml) of each culture were plated on CSM-His 2% Glu agar, and all plates were incubated at 30°C for 3 days. The frequency of Ty1*his3AI* retrotransposition is the number of His^+^ colonies divided by the total number of CFU plated on CSM-His 2% Glu agar, which was determined from the colony count on YPD agar. The average frequency and standard error for each genotype tested were calculated from nine separate cultures.

### Tunicamycin time course

A 250 ml culture of BY4741 harboring plasmid pLTR_p_:Gag_1–401_:GFP:*ADH1*_TER_ was grown to an OD_600_ of 0.25 at 20°C in CSM-Leu 2% Glu broth, and 250 µl of 5 mg/ml tunicamycin in DMSO (Research Products International, Corp) or DMSO only was added. Four OD_600_ units of cells were harvested 0, 1, 4, 8, and 18 hr after addition of tunicamycin. Lysates of each cell pellet were separated on 10% SDS-PAGE gels and analyzed by western blot analysis, as described above.

### FISH and fluorescence microscopy

Fluorescence *in situ* hybridization was performed essentially as described in Amberg et al. [72], with the following modifications. Strains were grown in YPD broth or, in the case of strains harboring plasmid pLTR_p_:Gag_1–401_:GFP:*ADH1*_TER_, CSM-Leu 2% Glu broth, to mid-log phase (OD_600_ of 0.4–0.6) at 20°C. For experiments with tunicamycin, a solution of tunicamycin in DMSO was added to a final concentration of 5 µg/ml, or an equivalent volume of DMSO was added, and cultures were grown for an additional 8 hr at 20°C. For experiments with cycloheximide, a solution of cycloheximide in DMSO was added to a final concentration of 0.44 µg/ml cycloheximide, or an equivalent volume of DMSO was added, and cultures were incubated with gentle turning at 20°C for 30 min. Cultures were treated with formaldehyde (4% final concentration) and incubated at room temperature for 15 min on a turning platform. Cells were collected by centrifugation, resuspended in 5 ml of 0.1 M KPO_4_ (pH 6.5)/4% formaldehyde, and rotated for 90 min at 23°C. Cells were washed twice with 0.1 M KPO_4_ (pH 6.5) and once in 1 ml wash buffer [0.1 M KPO_4_ (pH 6.5), 1.2 M sorbitol]. The cell pellet was resuspended in 1 ml wash buffer containing 500 µg of 100T Zymolyase (MP Biomedicals) and incubated for 30 min at 30°C. Spheroblasts were washed gently with wash buffer and resuspended in a volume of wash buffer approximately twice the volume of the pellet and transferred to 10-well slides (Electron Microscopy Sciences) pretreated with 0.1% poly-L-lysine (Sigma). After aspirating non-adhered cells, adhered cells were washed twice with 100 µl of 2× SSC (1× SSC = 0.15 NaCl, 0.015 M sodium citrate) per well. Cells in each well were incubated with 12 µl of prehybridization buffer (50% formamide, 10% dextran sulfate, 4× SSC, 0.02% polyvinyl pyrrolidone, 0.02% bovine serum albumin, 0.02% Ficoll-400, 125 µg/ml of tRNA, 500 µg/ml of denatured salmon sperm DNA) at 37°C for 1 hr in a humid chamber. Subsequently, 0.75 pmol of a Cy3-labeled *gag* anti-sense oligomer, PJ798 (5′ -/Cy3/TCT GTT TTG GAA GCT GAA ACG TGT AAC GGA TCT TGA TTT GTG TGG ACT TC - 3′), obtained from Integrated DNA Technologies, Inc., was added to each well, and slides were incubated for 12–18 hr at 37°C in a dark humid chamber. Slides were washed in 2× SSC for 3 min, 1× SSC for 5 min, 1× SSC containing 2 mg/ml DAPI for 3 min, 1× SSC for 5 min and 0.5× SSC for 5 min. After drying, coverslips were adhered to the slides with antifade solution [1.2% Mowiol-488 (Sigma), 3% glycerol, 50 mM Tris HCl (pH 8.5)]. Cells were visualized by fluorescence microscopy using a Zeiss Axioskop 200 M inverted microscope equipped with filter set: 31 (Cy3), 34 (DAPI) and 38 EX Band Pass 470/40 (GFP), at a magnification of 63 or 100×. A Q Imagining camera (or Hamamatsu ORCA ER) was used to obtain the images, which were then colored and merged in Openlab 4.0.4 software (Improvision) and modified with Photoshop CS software.

### Pho8-Ura3 translocation assay

Strains harboring plasmid pMP234 [29] were grown overnight in CSM-Leu 2% Glu broth at 20°C to an OD_600_ of 0.6 to 1.0. The OD_600_ of each culture was adjusted to 0.5 by addition of CSM-Leu 2% Glu broth. A 6 µl aliquot of each culture, and 10-fold serial dilutions of each culture in water, were spotted onto selective media containing glucose and grown at 20°C for 4 to 7 days.

## Supporting Information

Figure S1 Ty1 Gag isoforms present at steady-state in strain BY4741 do not correspond to unprocessed p49-Gag. Western blot analysis of Gag in wild-type strain BY4741, or the *spt3Δ* derivative expressing plasmid pGTy1-H3(pr-1682) [72], a *GAL1*-driven Ty1 element harboring a protease active-site mutation that blocks processing of p49-Gag. Anti-VLP polyclonal antibody was used to detect Gag.
(TIF)

Figure S2 Ty1 RNA, but not Gag, is associated with SRP-RNC complexes, and Ty1 retrosomes fail to form in an *rpl7aΔ* mutant. (A) RT-PCR analysis of RNA co-purified with affinity-purified TAP complexes from the *SRP54-TAP* (WT) and *SRP54-TAP rpl7aΔ* (*rpl7aΔ*) derivatives of strain BY4741 (top panel) or the *SRP54-TAP* (WT) and *SRP54-TAP bud21Δ* (*bud21Δ*) derivative of strain BY4741 (bottom panel). RNA isolated from cells treated with cycloheximide before TAP purification (Total RNA) or after purification of Srp54-TAP complexes (Srp54-TAP/TEV eluate) was analyzed by RT-PCR with gene-specific primers. Amplification was performed for 29 and 32 cycles (indicated by wedge). Reverse transcriptase was omitted from the cDNA synthesis reaction as a negative control (No RT). 7SL RNA, 18S rRNA and Y′ RNA were detected as positive controls for purification of SRP-RNC complexes. (B) Western blot analysis of whole cell lysate (WCL) using anti-CBP polyclonal antibody to detect Srp54-TAP and anti-VLP polyclonal antibody to detect Gag, and Srp54-TAP-purified complexes (TEV eluate) using anti-VLP antibody to detect Gag. (C) FISH analysis of Ty1 RNA and direct visualization of Gag∶GFP in cells of strain BY4741 (WT) and a congenic *rpl7aΔ* derivative, both harboring plasmid pLTR_p_:Gag_1–401_:GFP:*ADH1*_TER_. Cells were visualized by DIC (differential interference contrast) microscopy and fluorescence microscopy. DAPI (blue) stained nuclei. Ty1 RNA (red) was detected using a Cy3-labeled *gag* anti-sense probe. Gag∶GFP (green) was visualized directly. **f/c** is the Ty1 RNA foci per DAPI-stained cell and **n** is the total number of DAPI stained cells counted.
(TIF)

Figure S3 *DAmP* alleles of SRP, SRP receptor, ER translocon and Sec63 complex genes have varied defects in co-translational translocation of Pho8-Ura3 to the ER. Ten-fold serial dilutions of each strain (genotype indicated) harboring *LEU2*-based plasmid pMP234 expressing the Pho8-Ura3 reporter protein, were spotted onto CSM-Leu medium to monitor growth, CSM-Leu+5-FOA to measure 5-fluoororotic acid resistance (FOA^R^) and CSM-Ura-Leu medium to gauge Ura3 levels. CSM-Leu plates were incubated for 4 days at 20°C, while CSM-Leu+5-FOA and CSM-Ura-Leu were incubated for 7 days at 20°C. Pho8-Ura3 is a fusion of the N-terminal signal anchor sequence of the type II integral membrane protein Pho8 to the complete Ura3 protein. SRP-dependent translocation of Pho8-Ura3 to the ER confers a Ura^−^ (FOA^R^) phenotype in wild-type strains because Ura3 is retained in the ER lumen sequestered from its cytosolic substrate. An FOA-sensitive Ura^+^ phenotype is indicative of a defect in translocation of Pho8-Ura3 to the ER, resulting in Pho8-Ura3 accumulation in the cytoplasm.
(TIF)

Table S1 Primers used in plasmid and strain construction.
(DOCX)

Table S2 Gene-specific primers used in RT-PCR.
(DOCX)
