# Supplementary figures and images for: Co-translational Localization of an LTR-Retrotransposon RNA to the Endoplasmic Reticulum Nucleates Virus-Like Particle Assembly Sites
Source: PLoS Genet. 2014 Mar 6;10(3):e1004219. doi: 10.1371/journal.pgen.1004219 (PMC3945221; doi:10.1371/journal.pgen.1004219)

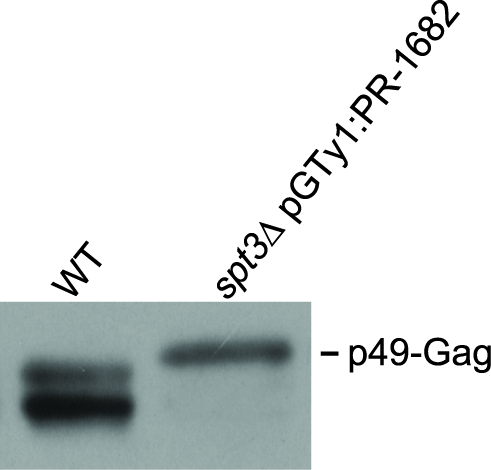

Supplement: Figure S1 — Ty1 Gag isoforms present at steady-state in strain BY4741 do not correspond to unprocessed p49-Gag. Western blot analysis of Gag in wild-type strain BY4741, or the spt3Δ derivative expressing plasmid pGTy1-H3(pr-1682) [72], a GAL1-driven Ty1 element harboring a protease active-site mutation that blocks processing of p49-Gag. Anti-VLP polyclonal antibody was used to detect Gag. (TIF) [file pgen.1004219.s001.tif]

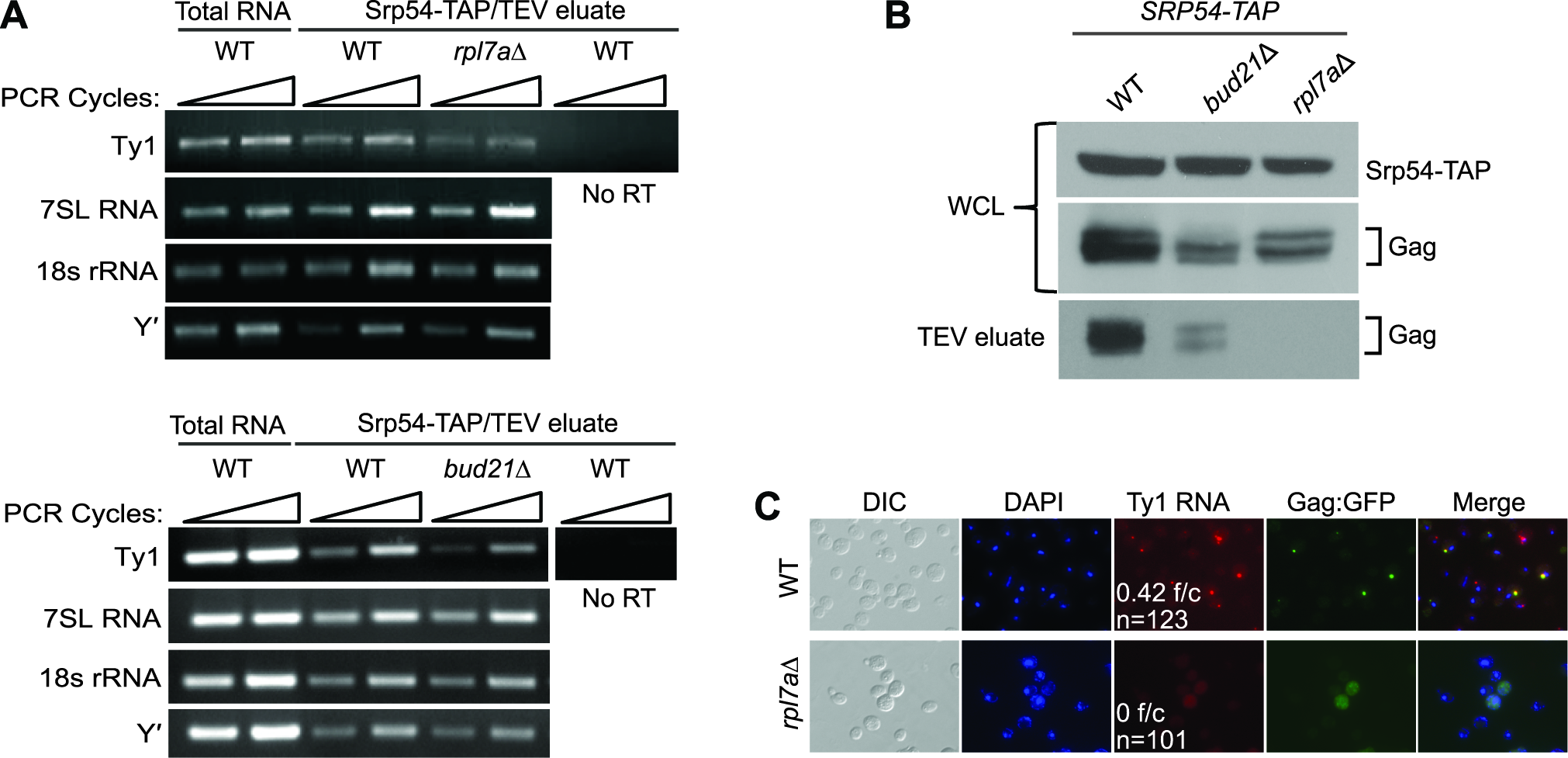

Supplement: Figure S2 — Ty1 RNA, but not Gag, is associated with SRP-RNC complexes, and Ty1 retrosomes fail to form in an rpl7aΔ mutant. (A) RT-PCR analysis of RNA co-purified with affinity-purified TAP complexes from the SRP54-TAP (WT) and SRP54-TAP rpl7aΔ (rpl7aΔ) derivatives of strain BY4741 (top panel) or the SRP54-TAP (WT) and SRP54-TAP bud21Δ (bud21Δ) derivative of strain BY4741 (bottom panel). RNA isolated from cells treated with cycloheximide before TAP purification (Total RNA) or after purification of Srp54-TAP complexes (Srp54-TAP/TEV eluate) was analyzed by RT-PCR with gene-specific primers. Amplification was performed for 29 and 32 cycles (indicated by wedge). Reverse transcriptase was omitted from the cDNA synthesis reaction as a negative control (No RT). 7SL RNA, 18S rRNA and Y′ RNA were detected as positive controls for purification of SRP-RNC complexes. (B) Western blot analysis of whole cell lysate (WCL) using anti-CBP polyclonal antibody to detect Srp54-TAP and anti-VLP polyclonal antibody to detect Gag, and Srp54-TAP-purified complexes (TEV eluate) using anti-VLP antibody to detect Gag. (C) FISH analysis of Ty1 RNA and direct visualization of Gag∶GFP in cells of strain BY4741 (WT) and a congenic rpl7aΔ derivative, both harboring plasmid pLTRp:Gag1–401:GFP:ADH1 TER. Cells were visualized by DIC (differential interference contrast) microscopy and fluorescence microscopy. DAPI (blue) stained nuclei. Ty1 RNA (red) was detected using a Cy3-labeled gag anti-sense probe. Gag∶GFP (green) was visualized directly. f/c is the Ty1 RNA foci per DAPI-stained cell and n is the total number of DAPI stained cells counted. (TIF) [file pgen.1004219.s002.tif]

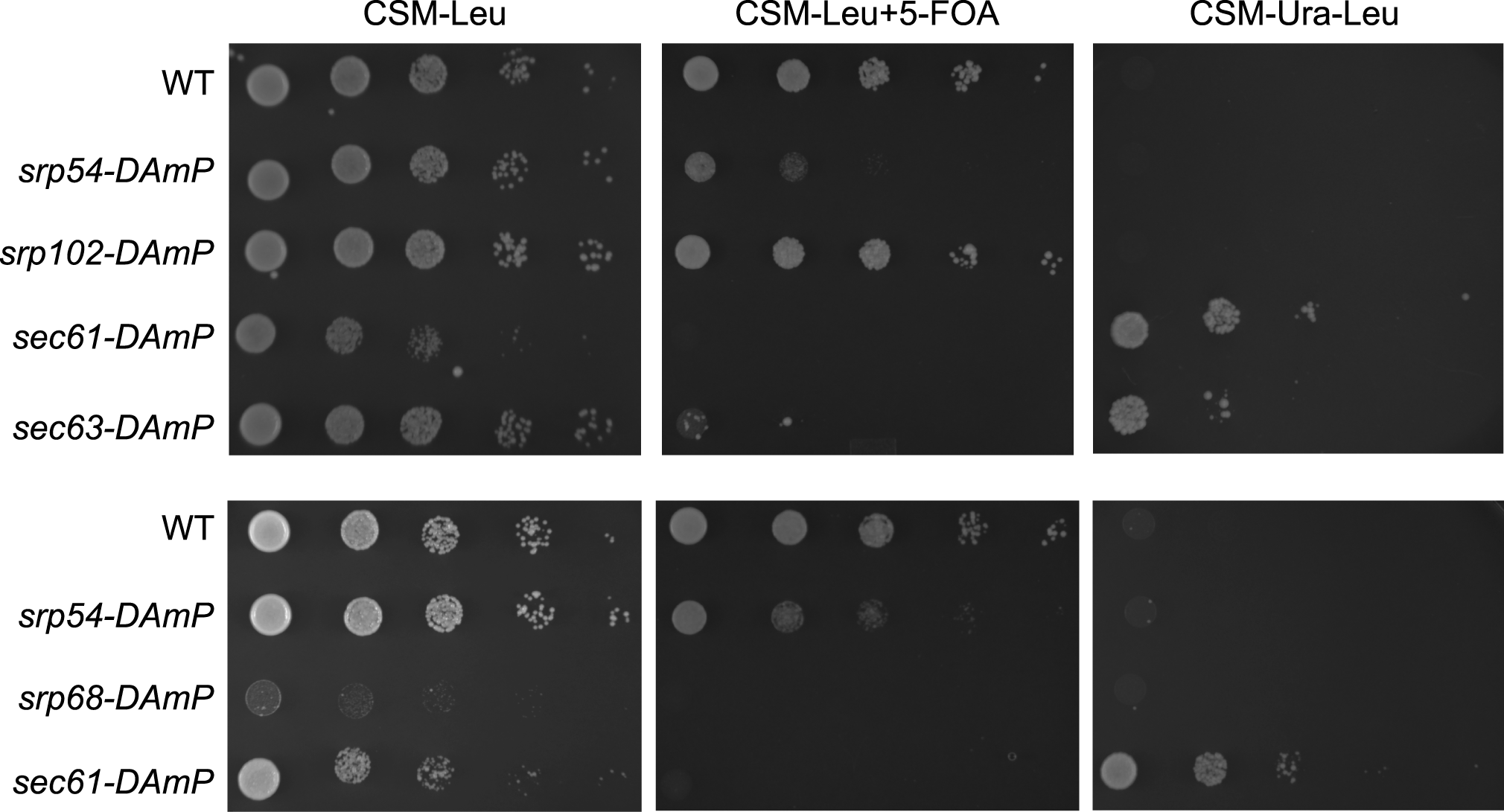

Supplement: Figure S3 — DAmP alleles of SRP, SRP receptor, ER translocon and Sec63 complex genes have varied defects in co-translational translocation of Pho8-Ura3 to the ER. Ten-fold serial dilutions of each strain (genotype indicated) harboring LEU2-based plasmid pMP234 expressing the Pho8-Ura3 reporter protein, were spotted onto CSM-Leu medium to monitor growth, CSM-Leu+5-FOA to measure 5-fluoororotic acid resistance (FOAR) and CSM-Ura-Leu medium to gauge Ura3 levels. CSM-Leu plates were incubated for 4 days at 20°C, while CSM-Leu+5-FOA and CSM-Ura-Leu were incubated for 7 days at 20°C. Pho8-Ura3 is a fusion of the N-terminal signal anchor sequence of the type II integral membrane protein Pho8 to the complete Ura3 protein. SRP-dependent translocation of Pho8-Ura3 to the ER confers a Ura− (FOAR) phenotype in wild-type strains because Ura3 is retained in the ER lumen sequestered from its cytosolic substrate. An FOA-sensitive Ura+ phenotype is indicative of a defect in translocation of Pho8-Ura3 to the ER, resulting in Pho8-Ura3 accumulation in the cytoplasm. (TIF) [file pgen.1004219.s003.tif]
