# Supplementary material for: Co-translational Localization of an LTR-Retrotransposon RNA to the Endoplasmic Reticulum Nucleates Virus-Like Particle Assembly Sites
Source: PLoS Genet. 2014 Mar 6;10(3):e1004219. doi: 10.1371/journal.pgen.1004219 (PMC3945221; doi:10.1371/journal.pgen.1004219)
Supplement: Table S1 — Primers used in plasmid and strain construction. (DOCX) [file pgen.1004219.s004.docx]

Table S1. Primers used in plasmid and strain construction

| Primer Name | Primer Sequence (5' to 3') |
| --- | --- |
| PJ762 | GCAATGGGCCCTGTTGGAATAGAAATCAA |
| PJ763 | CTAGAAGTTCTCCTCGAGGATTTAGG |
| PJ1205 | TGCATACATACATATATATATATACTGTGTATATTCAAATGGTGGCCTTTAATCGATGAATTCGAGCTCG |
| PJ1206 | TGATGCAAGATCCACAAATGCAACAAATGGCAAAACAATTCGGTATGGGCCGGATCCCCGGGTTAATTAA |
| PJ1207 | CAGAAGAGAAGGGAAAGAAAACACAGCATCGAGTCTAACTAAAGGAGATTGTACTGAGAGTGCAC |
| PJ1208 | ATACATACATATATATATATACTGTGTATATTCAAATGGTGGCCTCTGTGCGGTATTTCACACCG |
| PJ1231 | CAAAAGTGACTCCACAAATGATGC |
| PJ1232 | CTTTTCCATGGCAGTTCAATCGAA |
| PJ1233 | TGCTGCCCATTCCTAGTAAACCAA |
| PJ1234 | TAATTTAACGTATAGTTATGTAAA |
| PJ1235 | CCGCAGTTAGCAAAGTTCAAAAGA |
| PJ1236 | CTTGCGTTATCTCTCACAATGTAT |
| PJ1237 | ATAAAAGCGAATTGTTCACTGCAA |
| PJ1238 | CAACGCGTCTGACTTCTAATCAGA |
| PJ1293 | ACCCCAGTCTCTATACTCTTC |
| PJ1294 | GAAACCGCCATTGCTGAAGAC |
