# Supplementary material for: Co-translational Localization of an LTR-Retrotransposon RNA to the Endoplasmic Reticulum Nucleates Virus-Like Particle Assembly Sites
Source: PLoS Genet. 2014 Mar 6;10(3):e1004219. doi: 10.1371/journal.pgen.1004219 (PMC3945221; doi:10.1371/journal.pgen.1004219)
Supplement: Table S2 — Gene-specific primers used in RT-PCR. (DOCX) [file pgen.1004219.s005.docx]

Table S2. Gene-specific primers used in RT-PCR

| Primer name | RNA target | Primer sequence (5' to 3') |
| --- | --- | --- |
| PJ587 | Ty1 RNA | GTGATGACAAAACCTCTTCCG |
| PJ852 | Ty1 RNA | CCTATTACATTATCAATCCTTGC |
| PJ1176 | 7SL RNA | GATAGTTCTCTATTCCGCACCG |
| PJ1177 | 7SL RNA | ACCAGACAGAGAGACGGATTC |
| PJ1256 | 18S rRNA | GGTAATCTTGTGAAACTCCGT |
| PJ1257 | 18S rRNA | GTCCAAATTCTCCGCTCTGAG |
| YHELP-1 | Y' RNA | GTATGGAGCAACTTGCGTGAATC |
| YSEQ-UP | Y' RNA | CTGCTCCTCAACTGTCGATG |
| PJ1244 | *KAR2* mRNA | TTCTGGTGCCGCTGATTATGACGA |
| PJ1245 | *KAR2* mRNA | ACATATAATGCATATATTTACGTA |
| PJ1213 | *HRD1* mRNA | TCACCGTAGAACAGCTACAAA |
| PJ1214 | *HRD1* mRNA | GTATGTGGCCACAAGGTAACC |
| PJ1211 | *YOS9* mRNA | TATCGTTACCTAATGGACATA |
| PJ1212 | *YOS9* mRNA | CTCTGTGAATTAAGTATGTTC |
